# JMJD6 modulates DNA damage response through downregulating H4K16ac independently of its enzymatic activity

**DOI:** 10.1038/s41418-019-0397-3

**Published:** 2019-07-29

**Authors:** Dawei Huo, Hao Chen, Yiming Cheng, Xin Song, Kai Zhang, Mulin Jun Li, Chenghao Xuan

**Affiliations:** 1 Tianjin Key Laboratory of Medical Epigenetics; Key Laboratory of Breast Cancer Prevention and Therapy (Ministry of Education); Department of Biochemistry and Molecular Biology, Tianjin Medical University, 300070 Tianjin, China; 2 Department of Pharmacology, Tianjin Medical University, 300070 Tianjin, China

**Keywords:** DNA, Epigenetics

## Abstract

The initiation and transduction of DNA damage response (DDR) occur in the context of chromatin, and modifications as well as the structure of chromatin are crucial for DDR signaling. How the profound chromatin alterations are confined to DNA lesions by epigenetic factors remains largely unclear. Here, we discover that JMJD6, a Jumonji C domain-containing protein, is recruited to DNA double-strand breaks (DSBs) after microirradiation. JMJD6 controls the spreading of histone ubiquitination, as well as the subsequent accumulation of repair proteins and transcriptional silencing around DSBs, but does not regulate the initial DNA damage sensing. Furthermore, JMJD6 deficiency results in promotion of the efficiency of nonhomologous end joining (NHEJ) and homologous recombination (HR), rapid cell-cycle checkpoint recovery, and enhanced survival after irradiation. Regarding the mechanism involved, we demonstrate that JMJD6, independently of its catalytic activity, interacts with SIRT1 and recruits it to chromatin to downregulate H4K16ac around DSBs. Our study reveals JMJD6 as a modulator of the epigenome around DNA lesions, and adds to the understanding of the role of epigenetic factors in DNA damage response.

## Introduction

Among many types of DNA lesions, DNA double-strand breaks (DSBs) are considered the most harmful, because DSBs can lead to malignant transformation [1, 2]. DSBs occur through replication-fork collapse, during the processing of interstrand cross-links, or following exposure to ionizing radiation (IR) [1, 2]. Detection and repair of DSBs are integral to genomic stability and cell survival [3]. Upon detection of DSBs, cells trigger the DNA damage response in the context of chromatin. Therefore, histone modifications and chromatin structure around DSBs play essential roles in DDR [4], and the degree and spreading distance of histone modifications around DSBs should be tightly monitored. For example, the spreading of γH2A.X and histone ubiquitination is both well controlled to insulate chromatin from DNA damage signaling [5–8]. However, how the epigenetic alterations are confined to the sites of DNA damage is still an outstanding conundrum, and needs to be further investigated.

JMJD6, which contains a Jumonji C (JMJC) domain, functions as an iron- and α-oxoglutarate-dependent histone arginine demethylase [9, 10] or hydroxylase [11], regulating gene transcription and RNA splicing [10, 11]. Whether JMJD6 participates in genomic stability regulation, and whether its enzymatic activity is involved, need to be explored. We report here that JMJD6, independently of its catalytic activity, plays important roles in controlling DDR signaling in cells.

## Materials and methods

### Cells and reagents

U2OS and MCF-7 cells were from ATCC (Manassas, VA, USA). Cells were cultured in Dulbecco’s modified Eagle’s Medium supplemented with 10% fetal bovine serum (HyClone, Logan, Utah, USA), at 37 °C in a humidified atmosphere with 5% CO_2_. Cell lines were authenticated by examination of morphology and growth characteristics. The antibodies used were γH2A.X antibodies (clone JBW301, 05-636) and RNF168 antibodies (ABE367) from Merck KGaA (Darmstadt, Germany); MDC1 antibodies (NB100-397) from Novus Biologicals (Littleton, USA); FK2 antibodies (PW8810) from Enzo Life Sciences (Farmingdale, New York, USA); anti-JMJD6 (277011) from Synaptic Systems (Goettingen, Germany); anti-53BP1 (4937) from Cell Signaling Technology (Danvers, MA, USA); anti-JMJD6 (ab65770) from Abcam Inc. (Cambridge, MA, USA); anti-BRCA1 (sc-6954) and anti-JMJD6 (sc-28348) from Santa Cruz Biotechnology (Santa Cruz, CA, USA); anti-FLAG (M2, F3165) and anti-β-actin (A1978) from Sigma-Alderich (St Louis, MO, USA). Fluorescein- (111-095-003 and 115-095-003) or rhodamine-conjugated (111-025-003 and 115-025-003) secondary antibodies were obtained from Jackson ImmunoResearch Laboratories (West Grove, PA, USA), and horseradish peroxidase-conjugated secondary antibodies (sc-2030 and sc-2031) were from Santa Cruz Biotechnology, respectively. 4,6-diamidino-2-phenylindole (DAPI) was purchased from Sigma-Aldrich. U2OS-DR-GFP and U2OS-EJ5-GFP [12, 13] cells were obtained from Dr Jeremy Stark (City of Hope).

### Confocal immunofluorescence microscopy

Cells grown on glass coverslips were exposed to IR, and 1 or 8 h later, coverslips were fixed with methanol or paraformaldehyde, washed with PBS, and blocked with 0.8% bovine serum albumin in PBS. Coverslips were incubated at 37 °C with the primary antibodies for 1 h and subsequently with the fluorescein- and/or rhodamine-conjugated secondary antibodies for another hour, followed by staining with 0.5 µg/ml of DAPI for 5 min. Coverslips were mounted with 90% glycerol in PBS and examined with an Olympus confocal fluorescence microscope. Images were analyzed with CellProfiler software.

### RNA interference

For siRNA-mediated silencing, JMJD6 siRNA-1 (GAGGGAACCAGCAAGACGA), JMJD6 siRNA-2 (GTGTGGTGAGGATAACGAT), BRD4 siRNA-1 (GUGAGUACCGUGAUGCUCA), and BRD4 siRNA-2 (CUGAUUACUAUAAGAUCAU) were transfected into cells using RNAimax (Invitrogen, Carlsbad, CA, USA) according to the manufacturer’s instruction. Scrambled siRNA (UUCUCCGAACGUGUCACGU) was used as a control. All siRNAs were synthesized by Sigma-Aldrich. The targeting sequences of siJMJD6-1, siJMJD6-2 and control siRNA were separately cloned into the pLL3.7 lentiviral vector which contains a *GFP* cassette according to the manufacturer’s instruction. The recombinant construct, together with three assistant vectors (pRRE, VSVG, and RSV/REV), was then transiently transfected into HEK293T cells. Viral supernatants were collected both 24 and 48 h later, clarified by filtration, and concentrated by ultracentrifugation. These lentiviruses which express efficient shRNAs targeting JMJD6 or control shRNAs were employed to infect U2OS cells.

### Subcellular fractionation

In brief, cells were resuspended at a concentration of 4 × 10^7^ cells/ml in buffer A (10 mM HEPES [pH 7.9], 10 mM KCl, 1.5 mM MgCl_2_, 0.34 M sucrose, 10% glycerol, 0.1% Triton X-100, 1 mM dithiothreitol, and protease inhibitor cocktail) on ice for 8 min, and nuclei were collected by centrifugation (5 min, 1300 g, 4 °C). The supernatant was further clarified by centrifugation (5 min, 20,000 g, 4 °C), and the supernatant was collected as the cytosolic fraction (C). The nuclei were washed once in buffer A, and lysed for 30 min on ice in buffer B (3 mM EDTA, 0.2 mM EGTA, 1 mM dithiothreitol, and protease inhibitor cocktail), and nuclear soluble fraction (N) and insoluble chromatin (P) were separated by centrifugation (5 min, 1700 g, 4 °C).

### Micrococcal nuclease sensitivity assay

Chromatin fractions acquired as described above were resuspended in MNase buffer (10 mM Tris, 10 mM KCl, and 1 mM CaCl_2_), and MNase (Sigma-Aldrich) was added. After incubation at 37 °C for 5 min, the reaction was stopped with EDTA (1 mM, final concentration). Then RNase A and protein K were added into the mixture for 6 h at 65 °C. DNA was column-purified (QIAquick Spin Kit; Qiagen, Valencia, CA) and analyzed by 2% agarose gel electrophoresis.

### HR/NHEJ reporter assay

In U2OS-EJ5-GFP cells, an I-SceI-induced DSB can be generated within a chromosomally integrated inactive *GFP* cassette, and the *GFP* cassette was restored through the repair of the DSB by NHEJ [12, 14]. In U2OS-DR-GFP cells, an I-SceI-induced DSB was generated in the upstream *SceGFP* cassette, followed by HR that uses the downstream homologous template (*iGFP*) to prime nascent DNA synthesis, restoring the *GFP*+ cassette [12]. U2OS-DR-GFP and U2OS-EJ5-GFP cells (1 × 10^5^) were first transfected with individual siRNAs specific for JMJD6. Twenty-four hours later, HA-I-SceI expression constructs or empty vectors were transfected into the U2OS reporter cells using Lipofectamine 2000 (Invitrogen, Carlsbad, CA, USA). GFP+ cells were quantified by flow cytometry using a Flow Cytometer (Becton Drive, Franklin Lakes, NJ, USA) 48 h after transfection. For the gain-of-function experiment, FLAG-JMJD6 or FLAG-mutant expression constructs were transfected into U2OS-DR-GFP and U2OS-EJ5-GFP cells together with HA-I-SceI expression constructs. GFP+ cells were quantified by flow cytometry 48 h after transfection.

### Colony-formation assay

Equal numbers (5000 cells) of U2OS and JMJD6-depleted U2OS cells were seeded in triplicate in six-well plates. The cells were exposed to different doses of IR and were grown for 7 days before staining. Cells were washed with ice-cold PBS and fixed with cold methanol for 10 min. The Methanol was then removed and replaced by the crystal violet solution (0.2%) for 10 min. The cells were washed with ddH_2_O and dried at room temperature before analysis.

### Chromatin immunoprecipitation (ChIP)

U2OS-DR-GFP cells were washed twice with PBS and cross-linked for 10 min with 1% formaldehyde. Then cells were rinsed twice with and collected into ice-cold PBS. Cells were pelleted and resuspended in lysis buffer (1% SDS, 10 mM EDTA, 50 mM Tris-HCl, pH 8.1, and 1× protease inhibitor cocktail), and sonicated for ten cycles with Max amplitude (H mode) (30 s on, 30 s off) using a water-bath sonicator (Fisher Sonic Dismembrator; Model 300) before centrifugation for 10 min. Then immunoprecipitation was performed using antibodies against γH2A.X, 53BP1, BRCA1, SIRT1, H4K16ac, or normal IgG as a control. The eluted DNA fragments were purified with a DNA purification kit (QIAquick Spin Kit). Primer pair used in ChIP assays was as follows: 5′-AACCATGTTCATGCCTTCTT-3′ (forward) and 5′-CCTCGTGGGTCTTCTACTTT-3′ (reverse).

### ChIP-seq data analysis

JMJD6 ChIP-seq data (GSM1249905) and the H4K16ac ChIP-seq data (GSM985134) were downloaded from Cistrome [15]. Heatmaps for these signals around 6292 JMJD6-binding peaks were plotted using ChAsE [16].

### Statistical analysis

Group data were presented as mean ± SD. Comparisons between two groups were made by Student’s unpaired two-tailed *t*-tests. *P* values < 0.05 were considered significant. Analyses were performed using the Microsoft Excel and GraphPad Prism V6.0.

## Results

### JMJD6 is recruited to DSBs, but does not influence the initial DNA damage signaling

**Fig. 1:**
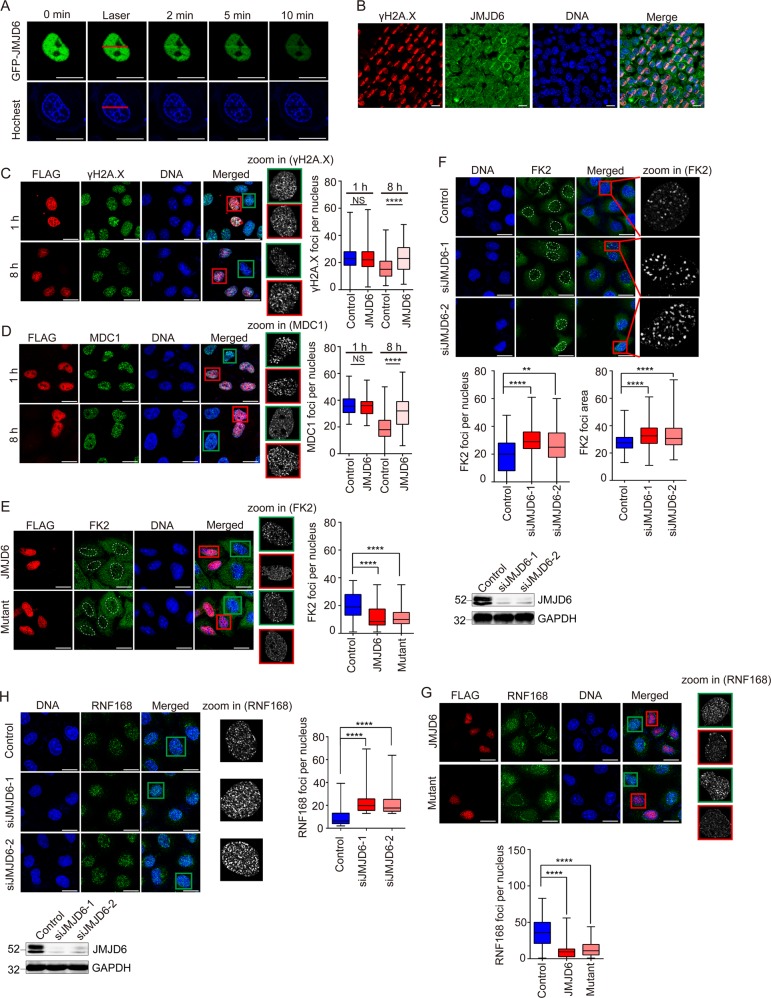
JMJD6 is recruited to DSBs, and limits the spreading of histone ubiquitination around DSBs independently of its enzymatic activity. **a** EGFP-JMJD6 is recruited to DNA damage sites. EGFP-JMJD6 expression constructs were transfected into U2OS cells, and the localization of EGFP-JMJD6 was observed under a fluorescence microscope following laser microirradiation. Scale bar, 20 μm. **b** The endogenous JMJD6 is recruited to laser irradiated regions. Cell treated with microirradiation were subjected to immunofluorescent staining using anti-JMJD6 together with anti-γH2A.X. Scale bar, 20 μm. **c** JMJD6 overexpression does not affect the initial γH2A.X foci formation, but prevents the vanishment of those foci. U2OS cells transfected with FLAG-JMJD6 expression constructs were treated with 10 Gy of IR, and immunofluorescence assays were performed using anti-FLAG together with anti-γH2A.X at 1 and 8 h after irradiation, respectively. Scale bar, 20 μm. **d** JMJD6 overexpression does not alter MDC1 foci formation, but prevents the vanishment of those foci. Cells were transfected with FLAG-JMJD6 expression constructs, exposed to 10 Gy of IR, and immunostained for FLAG and MDC1 at the indicated time. Scale bar, 20 μm. **e** JMJD6 overexpression inhibits the spreading of histone ubiquitination in response to IR. U2OS cells transfected with FLAG-JMJD6 or FLAG-mutant expression constructs were treated with 10 Gy of IR, and 1 h later, immunofluorescence assays were performed using anti-FLAG together with FK2 antibodies. Scale bar, 20 μm. **f** The spreading of histone ubiquitination in response to IR is increased upon depletion of endogenous JMJD6. U2OS cells transfected with JMJD6 siRNAs or control siRNAs were treated with 10 Gy of IR, and 1 h later, immunofluorescence assays were performed using FK2 antibodies. Scale bar, 20 μm. The knockdown effect induced by JMJD6 specific siRNAs was examined by western blot analysis using anti-JMJD6 and anti-GAPDH. **g** JMJD6 overexpression inhibits the spreading of RNF168 around DSBs. U2OS cells transfected with FLAG-JMJD6 or FLAG-mutant expression constructs were treated with 10 Gy of IR, and immunofluorescence assays were performed using anti-FLAG together with anti-RNF168. Scale bar, 20 μm. **h** JMJD6 knockdown increases the spreading of RNF168 around DSBs. Scale bar, 20 μm. The knockdown effect induced by JMJD6 specific siRNAs was examined by western blot analysis. For Fig. 1c–h, at least 50 nuclei of FLAG-JMJD6 expressing cells or control cells (cells without FLAG-JMJD6 expressing) from triplicate experiments were used to quantify the number of foci, and the *p*-value was determined by Student’s *t-*test. ****p < 0.0001, **p < 0.01, NS = not significant

JMJD6 was reported to modulate transcription and RNA splicing [10, 17]. However, whether JMJD6 regulates genomic stability has not been explored. To test whether JMJD6 is functionally involved in DDR, we first examined its distribution after DNA damage. We monitored the localization of EGFP-JMJD6 in response to laser microirradiation. EGFP and mCherry-PCNA proteins were used as the negative and positive control respectively. The results showed that EGFP-JMJD6, as well as mCherry-PCNA, was recruited to DNA damage sites (Fig. 1a, Supplementary Fig. 1), while EGFP could not be recruited to the laser sites (Supplementary Fig. 1). The endogenous JMJD6 was also recruited to laser irradiated regions (Fig. 1b). These data suggested that JMJD6 might be functionally involved in DDR.

DDR is initiated by the ATM-mediated phosphorylation of H2A.X, and the recruitment of MDC1 [18]. To test whether JMJD6 affects the initial DNA damage sensing, U2OS cells transfected with FLAG-JMJD6 expression constructs were treated with 10 Gy of IR, and immunofluorescence assays were performed using anti-FLAG together with anti-γH2A.X or anti-MDC1 at 1 and 8 h after irradiation, respectively. At 1 h after IR treatment, the γH2A.X and MDC1 foci formation was not affected by JMJD6 overexpression (Fig. 1c, d), demonstrating that JMJD6 overexpression did not affect initial DDR signaling. However, at 8 h after IR, the numbers of γH2A.X and MDC1 foci in JMJD6-overexpressed cells were significantly larger than that in the control cells without FLAG-JMJD6 overexpression (Fig. 1c, d), implying that JMJD6 overexpression intervened DNA repair, since as the DNA repair goes on, the foci should have become smaller/disappeared. Meanwhile, we found that JMJD6 overexpression did not change γH2A.X and MDC1 distribution in cells without IR treatment (Supplementary Fig. 2A, B). Furthermore, JMJD6 overexpression did not affect MDC1 expression, but increased the γH2A.X level only at 8 h after IR treatment (Supplementary Fig. 3), which is consistent with the results of immunofluorescence assays. These observations suggested that JMJD6 might regulate DDR by influencing the signaling cascade downstream of MDC1.

### JMJD6 limits the spreading of histone ubiquitination independently of its enzymatic activity

The recruitment of MDC1 is reported to generate a landing platform for RNF8 and RNF168 [19]. Once RNF168 is recruited, it spreads away from the DSB to amplify ubiquitin conjugates on K13/K15 of H2A [20, 21]. To examine whether JMJD6 regulates histone ubiquitination spreading, immunofluorescence assays were performed using anti-FLAG together with the antibody FK2 [5]. The results showed that overexpression of JMJD6 impaired accumulation of conjugated ubiquitin around DSBs (Fig. 1e). To test whether the enzymatic activity of JMJD6 is involved in this process, the proposed Fe(II)-binding residues in JMJD6 were substituted with nonchelating residues (H187A, D189A) to abolish both the histone demethylase and hydroxylase activity [9, 11]. JMJD6 enzymatic mutant overexpression decreased the spreading of histone ubiquitination just as the wild-type JMJD6 did (Fig. 1e), demonstrating that this process is independent of its enzymatic activity. Moreover, the size of FK2 foci, as well as its number, was larger upon depletion of endogenous JMJD6 (Fig. 1f), indicating an overspreading of histone ubiquitination from the damaged DNA in the absence of JMJD6. The changed histone ubiquitination detected by FK2 refers to ubiquitination on K13/K15 of H2A catalyzed by RNF168, since JMJD6 overexpression did not affect H2AK119ub distribution after IR treatment (Supplementary Fig. 4A). Consistently, JMJD6 or JMJD6 mutant overexpression inhibited the spreading of RNF168, while JMJD6 knockdown increased RNF168 recruitment around DSBs after IR treatment (Fig. 1g, h). However, JMJD6 overexpression did not affect the distribution of FK2 and RNF168 in cells without irradiation (Supplementary Fig. 2C, D). These data proved that JMJD6 controls the extension of RNF168-catalyzed H2A ubiquitination after IR treatment.

### JMJD6 controls the recruitment of repair proteins and the transcriptional silencing around DSBs

**Fig. 2:**
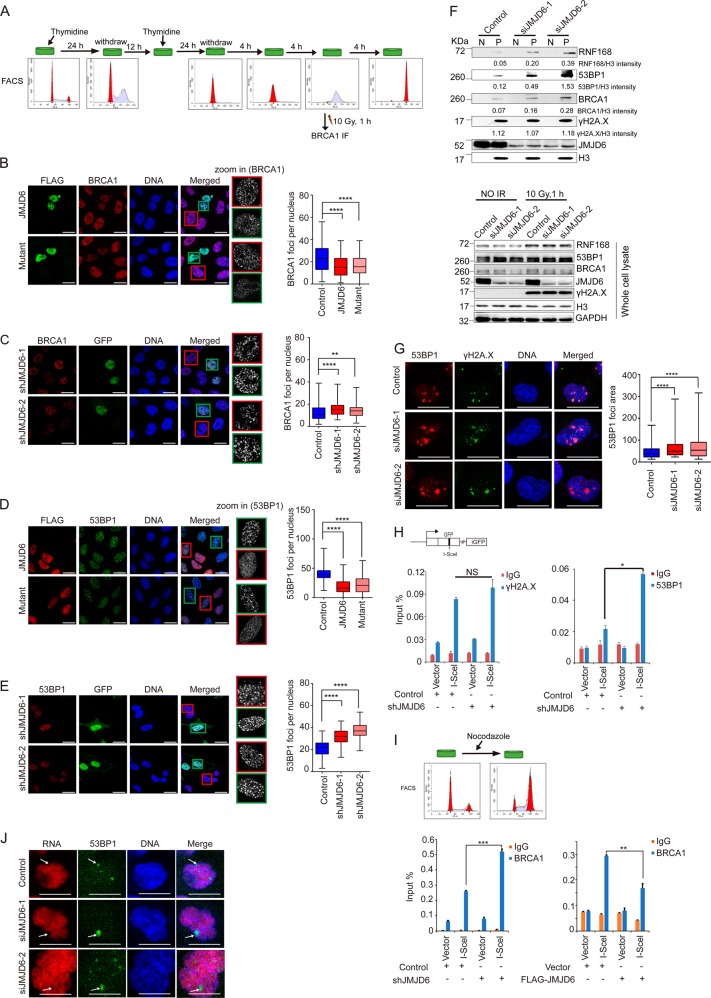
JMJD6 controls the accumulation of BRCA1 and 53BP1 as well as the transcriptional silencing around DSBs. **a** U2OS cells were synchronized by double thymidine treatment, and treated with IR at S phase. The cell cycle of the synchronized cells was detected by flow cytometry. **b** JMJD6 overexpression inhibits the accumulation of BRCA1 at DSBs in response to IR treatment. U2OS cells transfected with FLAG-JMJD6 or FLAG-mutant expression constructs were treated as shown in **a**, and 1 h later, immunofluorescence assays were performed using anti-FLAG together with anti-BRCA1. Scale bar, 20 μm. **c** JMJD6 knockdown leads to increased formation of BRCA1 foci after IR treatment. Synchronized U2OS cells were infected by lentiviruses containing JMJD6 shRNA sequences and a *GFP* cassette, treated with 10 Gy of IR, and 1 h later, immunofluorescence assays were performed using antibodies against BRCA1. Scale bar, 20 μm. **d** JMJD6 or its enzymatic mutant overexpression inhibits the accumulation of 53BP1 at DSBs in response to IR treatment. Scale bar, 20 μm. **e** JMJD6 knockdown leads to increased formation of 53BP1 foci after IR treatment. Scale bar, 20 μm. For Fig. 2b-e, at least 50 nuclei of FLAG-JMJD6 expressing cells or control cells (cells without FLAG-JMJD6 expressing) from triplicate experiments were used to quantify the number of foci, and the *p*-value was determined by Student’s *t-*test. ****p < 0.0001, **p < 0.01. **f** JMJD6 depletion results in increased association of RNF168 and downstream repair factors with chromatin. U2OS cells transfected with control or JMJD6 specific siRNAs were treated with 10 Gy of IR. Then the nuclear soluble fraction (N) and chromatin-bound proteins (P) were extracted and subjected to western blot analysis using antibodies against the indicated proteins. Total cell lysates were also subjected to western blot analysis. **g** Depletion of JMJD6 allows 53BP1 to spread beyond its physiological boundaries. U2OS cells were transfected with indicated siRNAs for 72 h and immunostained with the indicated antibodies 1 h after 0.25 Gy of IR treatment. Scale bar, 20 μm. **h** JMJD6 knockdown leads to increased accumulation of 53BP1 but no change of γH2A.X level near the DSB. U2OS-DR-GFP cells stably expressing control or JMJD6 shRNAs were transfected with empty vector or HA-I-SceI expression constructs. ChIP assays were performed using IgG, anti-γH2A.X or anti-53BP1, and the final DNA exactions were amplified by quantitative real-time PCR using primers that cover the DNA sequences near the I-SceI site. Each bar represents the mean ± S.D. for triplicate experiments and the *p*-value was determined by Student’s *t* test. *p < 0.05. **i** JMJD6 affects the accumulation of BRCA1 near the DSB. U2OS-DR-GFP cells transfected as indicated were treated with nocodazole; ChIP assays were performed using IgG or anti-BRCA1. Each bar represents the mean ± S.D. for triplicate experiments and the *p*-value was determined by Student’s *t-*test. ***p < 0.001, **p < 0.01. **j** Depletion of JMJD6 leads to unscheduled transcriptional silencing. U2OS cells transfected with the indicated siRNAs were treated with 0.25 Gy of IR, incubated in the presence of 5-ethinly uridin (5-EU) for the last 1 h, and then immunostained with anti-53BP1. The 5-EU incorporation to nascent mRNA was developed with Click-iT chemistry. Scale bar, 20 μm

RNF168-catalyzed H2A ubiquitination can recruit to the DSB-flanking chromatin genome caretakers including BRCA1 and 53BP1 [21, 22]. To detect the recruitment of BRCA1 which mediates subsequent HR repair, U2OS cells were synchronized in S phase, treated by IR, and immunofluorescence assays were performed 1 h later (Fig. 2a). JMJD6 as well as JMJD6 mutant overexpressing cells exhibited a remarkable reduction in BRCA1 foci formation (Fig. 2b), while in synchronized U2OS cells expressing JMJD6 shRNAs which were delivered via a lentiviral vector containing a *GFP* cassette, the accumulation of BRCA1 was significantly increased (Fig. 2c). Meanwhile, the enrichment of 53BP1 which mediates subsequent NHEJ repair was also examined after irradiation. JMJD6 or JMJD6 mutant overexpression led to a remarkable reduction in 53BP1 foci formation (Fig. 2d), and the accumulation of 53BP1 was significantly increased by JMJD6 knockdown (Fig. 2e). However, in U2OS cells without IR treatment, JMJD6 overexpression did not affect the distribution of BRCA1 and 53BP1 (Supplementary Fig. 2E, F). The inhibition of 53BP1 recruitment to DSBs by JMJD6 overexpression was also observed in IR-treated MCF-7 and A549 cells (Supplementary Fig. 4B), and this inhibition is JMJD6 specific, since FLAG-RBB (a transcriptional factor) overexpressing cells exhibited no change in 53BP1 foci formation after irradiation (Supplementary Fig. 4C).

In addition, the results of subcellular fractionation assays showed that JMJD6 depletion increased the association of RNF168, as well as 53BP1 and BRCA1 to chromatin upon IR treatment (Fig. 2f), without affecting total protein levels of these proteins (Fig. 2f). Meanwhile, western blot analysis showed that JMJD6 overexpression did not lead to decreased protein levels of RNF168, 53BP1, and BRCA1 (Supplementary Fig. 3), excluding the possibility that JMJD6 overexpression inhibits DDR effectors recruitment through downregulating their expressions. Furthermore, 53BP1 and γH2A.X were co-immunostained in control or JMJD6-depleted cells after IR treatment. The results showed that JMJD6 knockdown resulted in increased accumulation of 53BP1 without affecting the spreading of γH2A.X (Fig. 2g), indicating that JMJD6 knockdown uncouples histone phosphorylation and ubiquitination by allowing the latter to spread beyond its physiological boundaries. However, in cells without IR treatment, this phenomenon was not observed (Supplementary Fig. 2G). These data together demonstrated that JMJD6 limits the accumulation of repair proteins around IR-induced DSBs.

To further verify that JMJD6 modulates the recruitment of DNA repair proteins at DSBs, a cell-based system (DR-GFP) was used (Fig. 2h). In this system, a defective *GFP* cassette containing an I-SceI enzyme recognition site is stably incorporated into the genome, and a DSB can be generated by I-SceI expression [14]. After transfection of I-SceI expression constructs, γH2A.X and 53BP1 were detected to be enriched at the DSB proximal site (Fig. 2h), and JMJD6 knockdown resulted in no change of γH2A.X level but an increased recruitment of 53BP1 (Fig. 2h). Moreover, JMJD6 knockdown led to increased recruitment of BRCA1, and JMJD6 overexpression caused decreased BRCA1 enrichment around the DSB (Fig. 2i). The efficient knockdown by JMJD6 shRNAs was confirmed using real-time reverse transcription (RT)-PCR (Supplementary Fig. 5A).

Histone ubiquitination extension catalyzed by RNF168 around DSBs was reported to induce a silencing program in cis to DSBs to repress gene expression from a distant promoter [23]. By combining immunostaining of 53BP1 with in situ detection of nascent mRNA [5], we observed a significant reduction of de novo mRNA synthesis throughout the expanded chromatin domains in JMJD6-depleted cells under IR treatment (Fig. 2j), but this phenomenon could not be observed in cells without IR treatment (Supplementary Fig. 2H). These results indicated that the overexpansion of histone ubiquitination and the subsequent hyperaccumulation of repair proteins induced by JMJD6 depletion leads to pronounced transcriptional silencing around DSBs after IR treatment.

These results together demonstrated that JMJD6 regulates the magnitude of ubiquitin-dependent repair proteins accumulation and transcriptional silencing around DSBs.

### JMJD6 affects the efficiency of NHEJ and HR

**Fig. 3:**
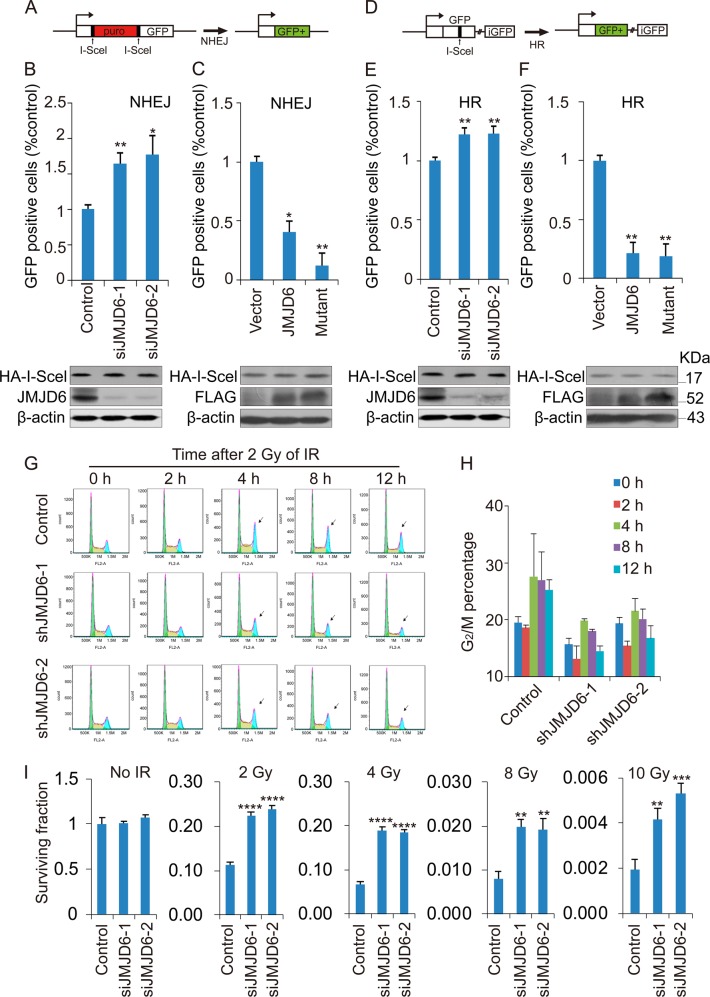
JMJD6 affects the efficiency of NHEJ and HR, as well as the cellular response to IR treatment. **a** Sketch map of NHEJ in U2OS-EJ5-GFP cells. **b** Depletion of JMJD6 leads to increased NHEJ efficiency. U2OS-EJ5-GFP cells were transfected with control or JMJD6 siRNAs, and 24 h later, HA-I-SceI expression constructs were transfected into these cells. The percentage of GFP+ cell was examined by FACS analysis 48 h after I-SceI transfection. The expression of JMJD6 and HA-I-SceI was determined by western blot analysis. **c** Overexpression of JMJD6 or its enzymatic mutant results in decreased NHEJ efficiency. U2OS-EJ5-GFP cells were transfected with empty vectors, FLAG-JMJD6 or FLAG-mutant expression constructs together with HA-I-SceI expression constructs, and 48 h later, the percentage of GFP+ cell was examined by FACS analysis. The expression of FLAG-JMJD6 and HA-I-SceI was determined by western blot analysis. **d** Sketch map of HR in U2OS-DR-GFP cells. **e** Depletion of JMJD6 leads to increased HR efficiency. **f** Overexpression of JMJD6 or its enzymatic mutant results in decreased HR efficiency. **g** JMJD6 depletion leads to a more rapid and efficient recovery from cell-cycle arrest after irradiation. U2OS cells stably expressing JMJD6 or control shRNAs were collected at indicated times after 2 Gy of IR treatment, and then subjected to propidium iodide staining and flow cytometry. **h** Cylindrical graphs presenting the change of percentage of G_2_/M cells detected by flow cytometry in figure G. **i** JMJD6 knockdown allows increased cell survival after IR treatment. Cell survival after irradiation in control or JMJD6 knockdown cells was measured by colony formation. Each bar represents the mean ± S.D. for triplicate experiments and the *p*-value was determined by Student’s *t-*test. ****p < 0.0001, ***p < 0.001, **p < 0.01, *p < 0.05

Then we validated these biochemical analyses by monitoring cellular parameters associated with DNA repair. We first examined the effect of JMJD6 overexpression or knockdown on the efficiency of two major DSB repair pathways, NHEJ and HR. U2OS-EJ5-GFP cell line was used to examine the efficiency of NHEJ through calculating percentage of GFP+ cells by flow cytometry (Fig. 3a). Our results manifested that depletion of JMJD6 by its specific siRNAs correlated with a significantly increased percentage of GFP+ cells (Fig. 3b), while overexpression of JMJD6 or its enzymatic mutant led to a markedly reduced percentage of GFP+ cells (Fig. 3c), indicating that JMJD6 regulates the efficiency of NHEJ repair. U2OS-DR-GFP cells were used to examine HR repair efficiency (Fig. 3d). The results showed that JMJD6 knockdown led to a significantly increased percentage of GFP-positive cells (Fig. 3e). Meanwhile, JMJD6 or its mutant overexpression resulted in a remarkably reduced percentage of GFP-positive cells (Fig. 3f). HR happens at late S and G_2_ phase of the cell cycle. Since JMJD6 overexpression or knockdown did not affect cell-cycle distribution of U2OS-DR-GFP cells (Supplementary Fig. 6), our data could demonstrate that JMJD6 also modulates HR repair efficiency.

Then U2OS cells stably expressing control or JMJD6 shRNAs were treated with 2 or 4 Gy of IR, collected at different time points after irradiation, and subjected to flow cytometry. The results indicated that JMJD6 depletion (the knockdown efficiency was shown in Supplementary Fig. 5B) allowed more rapid and efficient recovery from cell-cycle arrest after irradiation (Fig. 3g, h, Supplementary Fig. 7). Furthermore, colony formation assays confirmed that JMJD6 knockdown leads to increased cell survival after irradiation (Fig. 3i). Together, our experiments demonstrated that JMJD6 affects the repair efficiency of DSBs, as well as the subsequent cell-cycle recovery and cell survival after irradiation.

### JMJD6 interacts with SIRT1 and recruits it to chromatin

**Fig. 4:**
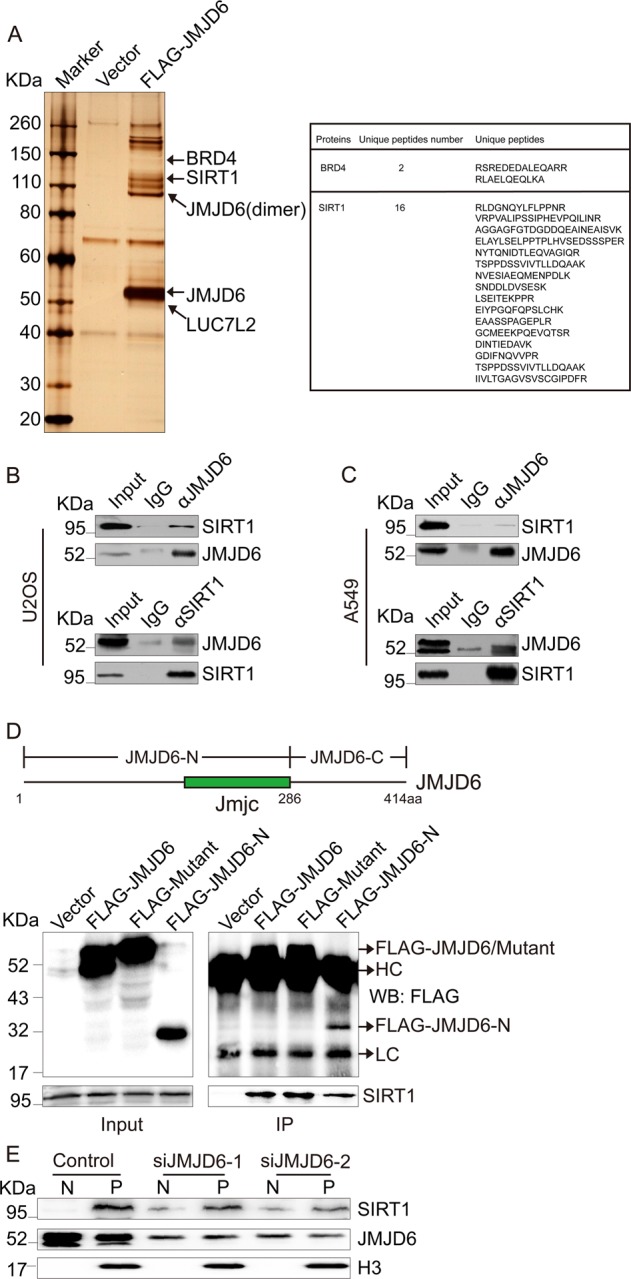
JMJD6 is required for the association of SIRT1 with chromatin. **a** Immunoaffinity purification of JMJD6-containing protein complexes. Cellular extracts from 293T cells expressing FLAG (vector) or FLAG-JMJD6 were immunopurified with anti-FLAG affinity columns and eluted with FLAG peptides. The eluates were resolved by SDS-PAGE and silver-stained. The protein bands were retrieved and analyzed by mass spectrometry. **b** JMJD6 interacts with SIRT1 in U2OS cells. Immunoprecipitation assays were performed with antibodies against the indicated proteins, followed by immunoblot analysis in U2OS cells. **c** JMJD6 interacts with SIRT1 in A549 cells. **d** The molecular detail of the interaction between JMJD6 and SIRT1. Whole-cell lysates from U2OS cells transfected with empty vector, FLAG-JMJD6, FLAG-mutant, or FLAG-JMJD6-N expression constructs were prepared, and immunoprecipitation was performed with anti-FLAG, followed by immunoblot analysis with indicated antibodies. **e** JMJD6 is required for the recruitment of SIRT1 to chromatin. The nuclear-soluble fraction (N) and chromatin-bound proteins (P) of U2OS cells, transfected with control or JMJD6-specific siRNAs, were extracted, and western blot analysis was performed using antibodies against the indicated proteins

To explore the mechanisms underlying JMJD6-mediated histone ubiquitination limitation around DSBs, we speculated that interactions between JMJD6 and other proteins probably accounted for the regulatory effects we observed. To test this, we applied affinity purification and mass spectrometry to identify proteins that potentially interact with JMJD6 in vivo. The lysates of 293T cells expressing FLAG-JMJD6 were prepared and subjected to FLAG affinity purification. The eluates were resolved on SDS-PAGE and silver-stained (Fig. 4a). Mass spectrometric analysis of the resolved protein bands showed that besides BRD4 and LUC7L2 which were previously reported to interact with JMJD6 [10, 11], the histone deacetylase SIRT1 was also co-purified with JMJD6 (Fig. 4a and Supplementary Fig. 8).

To validate affinity purification results, total proteins from U2OS cells were extracted and subjected to co-immunoprecipitation using antibodies against endogenous proteins. The results manifested that JMJD6 interacted with SIRT1 in U2OS cells (Fig. 4b). The interaction was also confirmed in A549 cells (Fig. 4c). To illustrate the molecular detail involved, co-immunoprecipitation assays were performed in U2OS cells expressing FLAG (vector), FLAG-JMJD6, FLAG-mutant, or FLAG-JMJD6-N (N-terminal fragment containing the JMJC domain, 1-286 aa) using anti-FLAG (FLAG-JMJD6-C expression constructs were not applied because the expression of C-terminal fragment was too low to be detected). The results showed that JMJD6 as well as its enzymatic mutant, and the N-terminal fragment, were all able to interact with SIRT1 (Fig. 4d).

JMJD6 has the lysine hydroxylase activity [11]. To test whether SIRT1 is a substrate of JMJD6, recombinant GST-SIRT1 purified from bacteria was used as the substrate and FLAG-JMJD6 purified from FLAG-JMJD6-expressing 293T cells was supplied as the hydroxylase. In vitro hydroxylation assay was performed, and then the mixture was resolved on SDS-PAGE and Coomassie blue-stained (Supplementary Fig. 9). The protein bands representing GST-SIRT1 on the gel were retrieved and analyzed using liquid chromatography-tandem mass spectrometry (LCMS/MS). The results showed that the addition of FLAG-JMJD6 in the reaction system did not result in an apparent lysine hydroxylation on GST-SIRT1 (Supplementary Table 1 and 2). This is consistent with our observation that JMJD6 modulates DDR independently of its enzymatic activity.

To further detect whether JMJD6 affects the localization of SIRT1, subcellular fractionation were performed in control or JMJD6-depleted cells. The results showed that JMJD6 knockdown led to the disassociation of SIRT1 from chromatin (Fig. 4e), indicating the essential role of JMJD6 for the association of SIRT1 with chromatin.

### JMJD6 regulates the H4K16ac level in cells

**Fig. 5:**
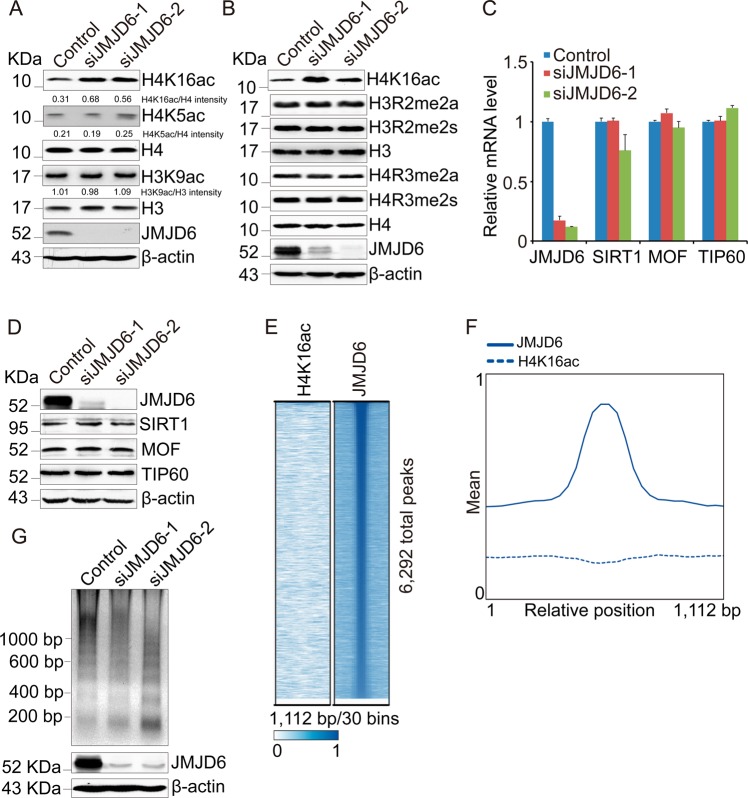
JMJD6 regulates the level of H4K16ac in cells. **a** Depletion of JMJD6 leads to increased H4K16ac level. Total proteins from U2OS cells transfected with the control or JMJD6 siRNAs were extracted, and western blot analysis was performed using antibodies against the indicated proteins. **b** JMJD6 knockdown does not increase the total level of methylation on H4R3 or H3R2. Total proteins from U2OS cells transfected with the control or JMJD6 siRNAs were extracted, and western blot analysis was performed using antibodies against the indicated proteins. **c** JMJD6 depletion does not change the mRNA levels of TIP60, MOF, or SIRT1. Total mRNA from U2OS cells transfected with indicated siRNAs was extracted, and quantitative real-time RT-PCR assays were performed. Each bar represents the mean ± S.D. for triplicate experiments. **d** JMJD6 knockdown does not change the protein level of TIP60, MOF, or SIRT1. **e** ChIP-seq density heatmaps of JMJD6 and H4K16ac around 6292 JMJD6-binding peaks in HeLa cells. **f** ChIP-seq profiling of JMJD6 and H4K16ac in HeLa cells over a 1112-bp window. **g** Knockdown of JMJD6 increases digestion by micrococcal nuclease. Nucleosomes from control or JMJD6-depleted U2OS cells were digested by micrococcal nuclease and then subjected to DNA gel electrophoresis

SIRT1 is responsible for deacetylating H4K16ac which is an essential histone modification mediating chromatin relaxation in DNA damage repair [24, 25]. Therefore, our finding that JMJD6 recruits SIRT1 to chromatin encouraged us to investigate whether JMJD6 regulates H4K16ac level. The results of western blotting manifested that JMJD6 depletion led to a significant increase in H4K16ac level, but did not change the levels of H4K5ac, H3K9ac, and total histones (Fig. 5a). Meanwhile, overexpression of JMJD6 or its catalytic mutant decreased H4K16ac level in cells (Supplementary Fig. 10). However, JMJD6 knockdown did not increase the global levels of H4R3me2s, H4R3me2a, H3R2me2s, and H3R2me2a (Fig. 5b), which ruled out the possibility that the increase of H4K16ac induced by JMJD6 knockdown was mediated by regulating methylation on H4R3 or H3R2 at the genomic level.

The acetylation of H4K16 is catalyzed by TIP60 and MOF, whereas it is mainly removed by SIRT1 [25–27]. To exclude the possibility that the upregulation of H4K16ac by JMJD6 depletion might also be mediated by changing the expression of these three enzymes, real-time RT-PCR analysis and western blot analysis were performed in JMJD6-depleted cells. The results showed that JMJD6 knockdown did not affect the mRNA and protein levels of TIP60, MOF, and SIRT1 (Fig. 5c, d).

To further inspect the genome-wide relationship between the binding of JMJD6 to chromatin and the acetylation of H4K16 in cells, we obtained JMJD6 ChIP-seq data (GSM1249905) [10] and H4K16ac ChIP-seq data (GSM985134) in HeLa cells from Cistrome [15]. We plotted heatmaps for these signals around 6292 JMJD6-binding peaks using ChAsE [16]. The results manifested that, in general, H4K16ac is depleted from JMJD6-binding sites (Fig. 5e, f).

Since the acetylation of histone H4K16 is linked to relaxed chromatin structure [28], to further detect the effect of JMJD6 knockdown on global chromatin structure, micrococcal nuclease susceptibility experiments were performed. The results showed that knockdown of JMJD6 increased digestion by micrococcal nuclease (Fig. 5g), implying that JMJD6 depletion is related to a more “open” overall chromatin structure.

Taken together, our results demonstrated that JMJD6, which is essential for the chromatin recruitment of SIRT1, negatively regulates cellular H4K16ac level.

### JMJD6 modulates the H4K16ac level around DSBs

**Fig. 6:**
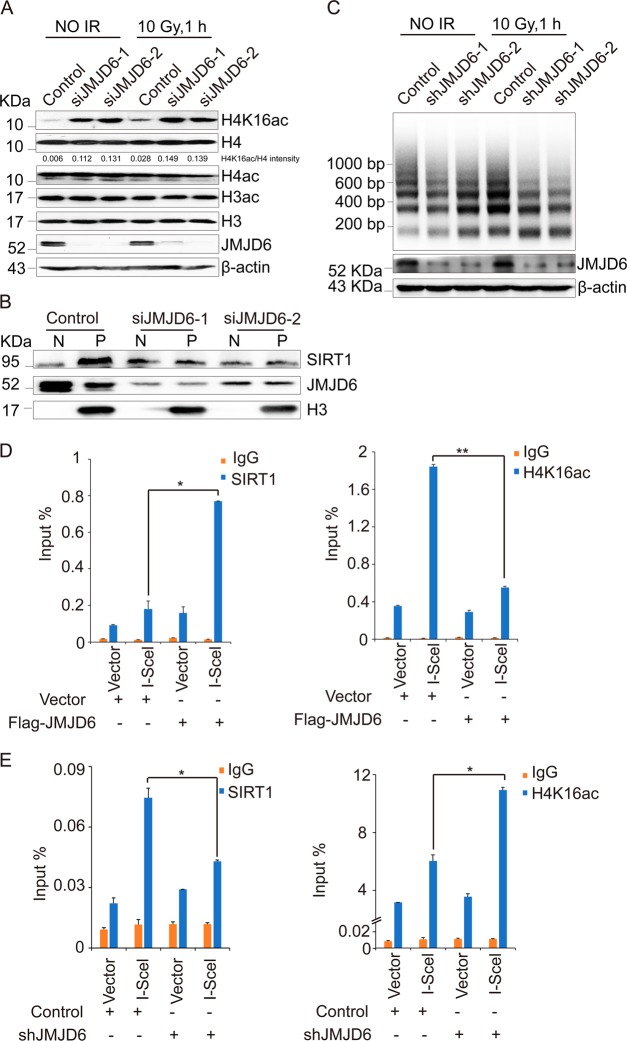
JMJD6 modulates the H4K16ac level around DSBs. **a** Depletion of JMJD6 increases H4K16ac in U2OS cells after ionizing radiation. U2OS cells transfected with control or JMJD6 siRNAs were untreated or treated with 10 Gy of IR, and 1 h later, the cell lysates were extracted and subjected to immunoblot analysis using indicated antibodies. **b** JMJD6 is required for the recruitment of SIRT1 to chromatin after IR treatment. U2OS cells transfected with control or JMJD6 specific siRNAs were treated with 10 Gy of IR, and 1 h later, the nuclear-soluble fraction (N) and chromatin-bound proteins (P) of U2OS cells were extracted and subjected to western blot analysis using antibodies against the indicated proteins. **c** Knockdown of JMJD6 increases digestion by micrococcal nuclease in response to IR treatment. Control or JMJD6-depleted U2OS cells were treated with IR or not. The nucleosomes were digested by micrococcal nuclease and subjected to DNA gel electrophoresis. **d** JMJD6 overexpression leads to increased recruitment of SIRT1 and decreased level of H4K16ac around the DSB. U2OS-DR-GFP cells were transfected with indicated expression constructs. ChIP assays were performed using IgG, anti-SIRT1 or anti-H4K16ac, and the final DNA exactions were amplified by quantitative real-time PCR using primers that cover the DNA sequences around the I-SceI site. Each bar represents the mean ± S.D. for triplicate experiments and the *p*-value was determined by Student’s *t-*test. **p < 0.01, *p < 0.05. **e** JMJD6 knockdown results in decreased recruitment of SIRT1 and increased level of H4K16ac around the DSB. U2OS-DR-GFP cells stably expressing control or JMJD6 shRNAs were transfected with empty vector or HA-I-SceI expression constructs. ChIP assays were performed using indicated antibodies. Each bar represents the mean ± S.D. for triplicate experiments and the *p*-value was determined by Student’s *t-*test. *p < 0.05

H4K16ac can extend for hundreds of kilobases away from DNA breaks [29], providing the accessibility of signaling molecules to DNA damage sites [30]. It has been reported that the impaired H4K16ac, caused by inactivating Trrap (the cofactor of TIP60), blocks RNF8/RNF168-catalyzed histone ubiquitination, leading to the inhibition of the subsequent loading of effector proteins onto chromatin, without affecting the initial DNA damage sensing [31]. This phenomenon is consistent with the consequence mediated by JMJD6 overexpression, suggesting that JMJD6-mediated limitation of histone ubiquitination is achieved by downregulating H4K16ac around DSBs. The results of western blotting confirmed that IR treatment increased H4K16ac level (Fig. 6a), and depletion of JMJD6 led to a remarkable increase in the level of H4K16ac but not total H4ac and H3ac after IR treatment (Fig. 6a). Consistently, in U2OS cells under IR treatment, JMJD6 interacted with SIRT1 (Supplementary Fig. 11A), and JMJD6 knockdown resulted in dissociation of SIRT1 from chromatin (Fig. 6b). Furthermore, the results of micrococcal nuclease susceptibility experiments showed that under IR treatment, knockdown of JMJD6 increased digestion by micrococcal nuclease (Fig. 6c). Besides, in U2OS-DR-GFP cells, JMJD6 overexpression increased the recruitment of SIRT1 to the DSB, thus decreasing the H4K16ac level around the DSB (Fig. 6d). While JMJD6 knockdown led to decreased recruitment of SIRT1, further raising the level of H4K16ac near the DSB (Fig. 6e). Our data indicated that JMJD6 downregulates H4K16ac around DSBs, which clarifies the mechanism underlying JMJD6-mediated limitation of DDR signaling.

### JMJD6-mediated DDR regulation is SIRT1- and BRD4 dependent

**Fig. 7:**
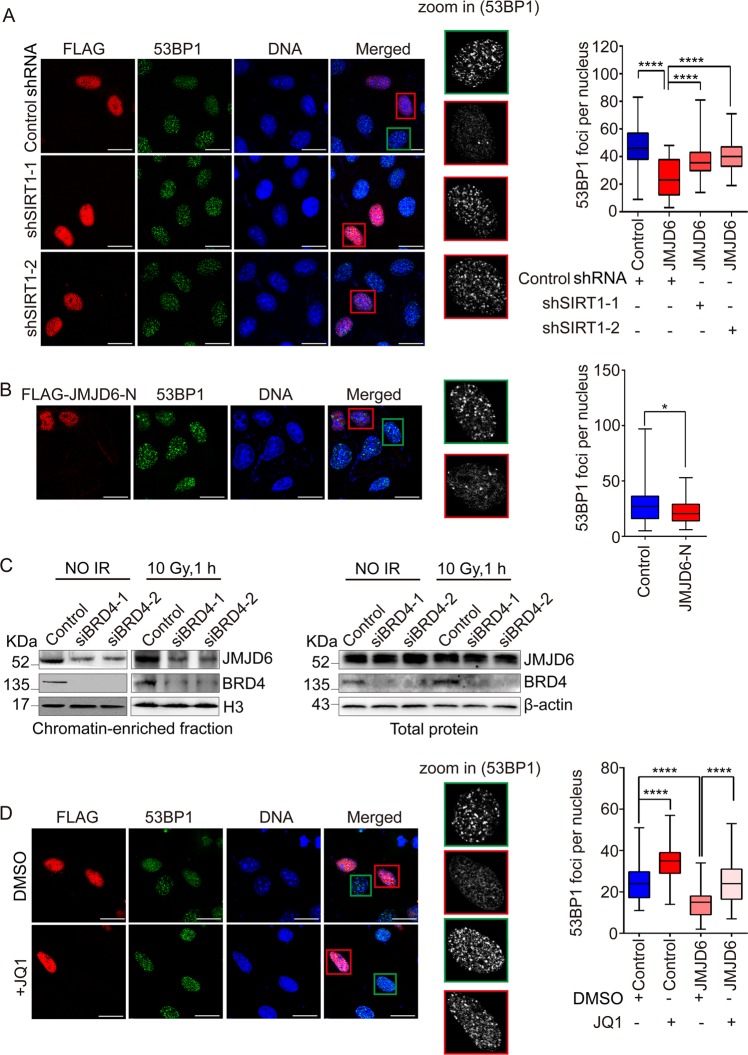
The impaired DDR mediated by JMJD6 overexpression is SIRT1- and BRD4 dependent. **a** SIRT1 knockdown counteracts the impaired 53BP1 foci formation in JMJD6-overexpressed cells. U2OS cells stably expressing shRNAs specific for SIRT1 or control shRNAs were transfected with FLAG-JMJD6 expression constructs, and immunofluorescence experiments were performed using anti-FLAG together with anti-53BP1 1 h after IR treatment. Scale bar, 20 μm. At least 50 nuclei of FLAG-JMJD6 expressing cells or control cells (cells without FLAG-JMJD6 expressing) from triplicate experiments were used to quantify the number of foci, and the *p*-value was determined by Student’s *t-* test. ****p < 0.0001. **b** Overexpression of FLAG-JMJD6-N inhibits the recruitment of 53BP1 to DSBs. U2OS cells transfected with FLAG-JMJD6-N expression constructs were treated with 10 Gy of IR, and 1 h later, immunofluorescence assays were performed using anti-FLAG together with anti-53BP1. Scale bar, 20 μm. *p < 0.05. **c** BRD4 is essential for the recruitment of JMJD6 to chromatin. U2OS cells transfected with control or BRD4 specific siRNAs were treated with or without IR. Chromatin-bound proteins or total proteins were extracted and then subjected to western blot analysis using indicated antibodies. **d** The impaired loading of 53BP1 in JMJD6-overexpressed cells is counteracted by BRD4 inhibition. U2OS cells transfected with FLAG-JMJD6 expression constructs were untreated or treated with JQ1, and 1 h after IR treatment, immunofluorescence experiments were performed using anti-FLAG together with anti-53BP1. Scale bar, 20 μm. At least 50 nuclei of FLAG-JMJD6 expressing cells or control cells (cells without FLAG-JMJD6 expressing) from triplicate experiments were used to quantify the number of foci, and the *p*-value was determined by Student’s *t-*test. ****p < 0.0001

To further investigate the role of SIRT1 in the impaired DDR in JMJD6-overexpressed cells. U2OS cells stably expressing shRNAs specific for SIRT1 or control shRNAs were transfected with FLAG-JMJD6 expression constructs, treated with IR or not, and immunofluorescence assays were performed. As shown in Fig. 7a and Supplementary Fig. 12A, SIRT1 knockdown (the efficiency was shown in Supplementary Fig. 5C) could abrogate the suppressive effect of JMJD6 overexpression on the accumulation of 53BP1 after IR treatment, demonstrating that SIRT1 is indispensable for JMJD6-mediated DDR regulation. In addition, overexpression of FLAG-JMJD6-N, which can interact with SIRT1, could also inhibit the recruitment of 53BP1 to DSBs after IR treatment (Fig. 7b, Supplementary Fig. 12B).

JMJD6 was previously reported to interact with BRD4 to coordinately regulate transcription in HeLa cells [10]. This interaction was also detected in our affinity purification assay (Fig. 4a), and was confirmed in normal and IR-treated U2OS cells by co-immunoprecipitation assays (Supplementary Fig. 11). To investigate whether BRD4, which directly binds to acetyl-lysine on histone [32], mediates the recruitment of JMJD6 to chromatin under IR treatment, chromatin-bound proteins from control or BRD4-depleted U2OS cells were extracted and subjected to western blot analysis. The results showed that the association of JMJD6 with chromatin was decreased upon BRD4 depletion (Fig. 7c), indicating the requirement of BRD4 for the recruitment of JMJD6 to chromatin in response to DNA damage.

BRD4 was recruited to damaged chromatin by IR treatment to inhibit overspreading of γH2A.X [8]. To discover the role of BRD4 in the impaired DDR induced by JMJD6 overexpression, JQ1, a small molecule inhibitor of BET bromodomains, was used to inhibit the chromatin binding of BRD4. The results of immunofluorescence assays showed that the impaired 53BP1 foci formation in JMJD6-overexpressed cells was counteracted by JQ1 treatment under IR treatment (Fig. 7d, Supplementary Fig. 12C), indicating the requirement of BRD4 for JMJD6-mediated regulation of DDR.

## Discussion

Cellular response to DSBs is a highly dynamic signaling pathway, which needs constant monitoring by inhibitory mechanisms to fine-tune the cellular response to DNA lesions in both space and time [33]. The negative regulation of the DSB response has been reported to occur at different points of DDR, for example, the dephosphorylation of γH2A.X by PP4 [34], the degradation of MDC1 mediated by RNF4 [35], the limitation of histone ubiquitination spreading by TRIP12- and UBR5-mediated degradation of RNF168 [5], and so on. Here, we demonstrate that JMJD6 controls DNA damage response through the negative regulation of H4K16ac around DSBs, revealing a novel negative regulatory mechanism during DDR. The modulation of DDR by JMJD6 is independent of its catalytic activity, making this interesting protein with multi-manner activities worthy of further investigation and validation.

It has been reported that oncogenes, such as *ras* and *E2F1*, can induce DSBs in tumor cells [36–38], leading to the genomic instability which characterizes the vast majority of human cancers [39]. In these cancer cells, DNA DSBs exist, but the DNA damage checkpoint pathway is compromised during cancer development, often by mutation/downregulation of checkpoint proteins [3, 36, 40]. Furthermore, inhibition of certain repair pathways would lead to a shift in repair mechanisms particularly to error-prone ones that facilitates genomic instability [41]. JMJD6 is upregulated in several types of cancer [42]; it is natural to speculate that JMJD6 overexpression-mediated inhibition of DNA repair may be one of the reasons for the increased genomic instability of tumor cells. Our study uncovers a novel function of JMJD6 in H4K16ac regulation and DNA damage response, and suggests a molecular mechanism for how overexpression of JMJD6 leads to increased genomic instability, thus promoting cancer development.

## Supplementary information

Supplementary Figures and Tables
