# Supplementary material for: JMJD6 modulates DNA damage response through downregulating H4K16ac independently of its enzymatic activity
Source: Cell Death Differ. 2019 Jul 29;27(3):1052–66. doi: 10.1038/s41418-019-0397-3 (PMC7206091; doi:10.1038/s41418-019-0397-3)
Supplement: Supplementary file 1 — Supplementary Figures and Tables [file 41418_2019_397_MOESM1_ESM.pdf]

Supplementary Figure Legends

Supplementary Figure 1. EGFP-JMJD6 is recruited to DNA damage sites. EGFP-JMJD6, EGFP, and mCherry-PCNA expression constructs were transfected into U2OS cells respectively, and the localization of these three proteins was observed under a fluorescence microscope following laser microirradiation. The fluorescence intensity was quantified using the software ZEN2.5 at 0.2 μm intervals in the range of 6 μm centered on the center of laser locus, and the fluorescence intensity curve was plotted according to the values of the scattered plots. About 80% of the cells showed significant EGFP-JMJD6 recruitment in response to laser treatment. Scale bar, 10 μm.

Supplementary Figure 2. JMJD6 overexpression does not change the distribution of DDR effectors in cells without IR treatment. FLAG-JMJD6 or FLAG-Mutant expression constructs were transfected in to U2OS cells, and immunofluorescence assays were performed using anti-FLAG together with anti-γH2A.X (A), anti-MDC1 (B), FK2 (C), anti-RNF168 (D), anti-53BP1 (E), or anti-BRCA1 (F). (G) U2OS cells were transfected with indicated siRNAs for 72 hours and immunostained with anti-γH2A.X together with anti-53BP1. (H) U2OS cells transfected with the indicated siRNAs were incubated in the presence of 5-ethinly uridin (5-EU) for the last 1 hour, and then immunostained with anti-53BP1. The 5-EU incorporation to nascent mRNA was developed with Click-iT chemistry. Scale bar in Supplementary Figure 2, 20 μm.

Supplementary Figure 3. JMJD6 overexpression does not decrease the protein

level of DNA damage response proteins. FLAG-JMJD6 or FLAG-Mutant expression constructs were transfected in to U2OS cells which were then treated with 10 Gy of IR or not. At 1 hour and 8 hours after IR treatment, cell lysates were collected and subjected to western blotting using indicated antibodies.

Supplementary Figure 4. The modulation of DDR by JMJD6 is specific and has no cell specificity. (A) JMJD6 overexpression does not affect the level of H2AK119ub after IR treatment. Immunofluorescence assays were performed using antibodies against FLAG together with H2AK119ub in FLAG-JMJD6 overexpressed U2OS cells at 1 hour after irradiation. Scale bar, 20 μm. (B) The impaired 53BP1 foci formation mediated by JMJD6 overexpression is cell type independent. MCF-7 cells and A549 cells transfected with FLAG-JMJD6 or FLAG-mutant expression constructs were treated with IR, and immunofluorescence assays were performed using anti-FLAG together with anti-53BP1. Scale bar, 20 μm. At least 50 nuclei from triplicate experiments for each group were quantified, and the *p*-value were determined by Student's *t* test. ****p < 0.0001. (C) The effect of JMJD6 overexpression on DDR is specific. We transfected FLAG-RBB expression constructs into U2OS cells, and examined foci formation of 53BP1 after IR treatment using immunofluorescence assays. Scale bar, 20 μm.

Supplementary Figure 5. The knockdown effect mediated by JMJD6 shRNAs and SIRT1 shRNAs. (A) The effect of JMJD6 knockdown by shRNAs in U2OS-DR-GFP

cells was detected by real-time RT-PCR. (B) Cell lysates of U2OS cells stably expressing JMJD6 shRNAs or control shRNAs were obtained and subjected to western blot analysis using indicated antibodies. (C) Cell lysates of U2OS cells stably expressing SIRT1 shRNAs or control shRNAs were subjected to western blot analysis using antibodies against SIRT1 and β-actin.

Supplementary Figure 6. JMJD6 overexpression or knockdown does not affect the cell cycle of U2OS-DR-GFP cells. U2OS-DR-GFP cells were transfected with control siRNAs, JMJD6 siRNAs, vector, FLAG-JMJD6, or FLAG-Mutant expression constructs, and cell cycle was analyzed by flow cytometry.

Supplementary Figure 7. JMJD6 depletion leads to more rapid and efficient recovery from cell cycle arrest after irradiation. U2OS cells stably expressing JMJD6 or control shRNAs were collected at indicated times after 4 Gy of IR treatment, and then subjected to propidium iodide staining and flow cytometry.

Supplementary Figure 8. The mass spectrometry detail of LUC7L2 protein.

Supplementary Figure 9. JMJD6 does not catalyze lysine hydroxylation on SIRT1. Recombinant GST-SIRT1 purified bacterially was incubated with control or FLAG-JMJD6 purified from FLAG-JMJD6-expressing 293T cells in the presence of 2OG, ascorbate, and FeNH4SO4, and then the mixture was resolved on SDS-PAGE and

Coomassie blue-stained. The protein bands representing GST-SIRT1 on the gel were retrieved and analyzed using LCMS/MS. The detailed result of LCMS/MS is supplied as Supplementary Table 1 and 2.

Supplementary Figure 10. Overexpression of JMJD6 and its catalytic mutant decreases H4K16ac level. The cell lysates from U2OS cells transfected with FLAG-JMJD6 or FLAG-mutant expression constructs were obtained and subjected to western blot analysis using indicated antibodies.

Supplementary Figure 11. JMJD6 interacts with BRD4 and SIRT1 in U2OS cells under IR treatment. (A) Immunoprecipitation assays were performed in U2OS cells treated with IR or not. (B) Immunoprecipitation assays were performed with antibodies against the indicated proteins followed by immunoblot analysis in U2OS cells.

Supplementary Figure 12. JMJD6 overexpression does not change the distribution of 53BP1 in cells without IR treatment. (A) U2OS cells stably expressing shRNAs specific for SIRT1 or control shRNAs were transfected with FLAG-JMJD6 expression constructs, and immunofluorescence experiments were performed using anti-FLAG together with anti-53BP1. Scale bar, 20 μm. (B) U2OS cells were transfected with FLAG-JMJD6-N expression constructs, and immunofluorescence assays were performed using anti-FLAG together with anti-53BP1. Scale bar, 20 μm. (C) U2OS cells transfected with FLAG-JMJD6 expression constructs were untreated or treated

with JQ1, and immunofluorescence experiments were performed using anti-FLAG together with anti-53BP1. Scale bar, 20 μm.

Supplementary Table 1. The detailed information of modifications on GST-SIRT1 which was incubated with control samples.

Supplementary Table 2. The detailed information of modifications on GST-SIRT1 which was incubated with FLAG-JMJD6.

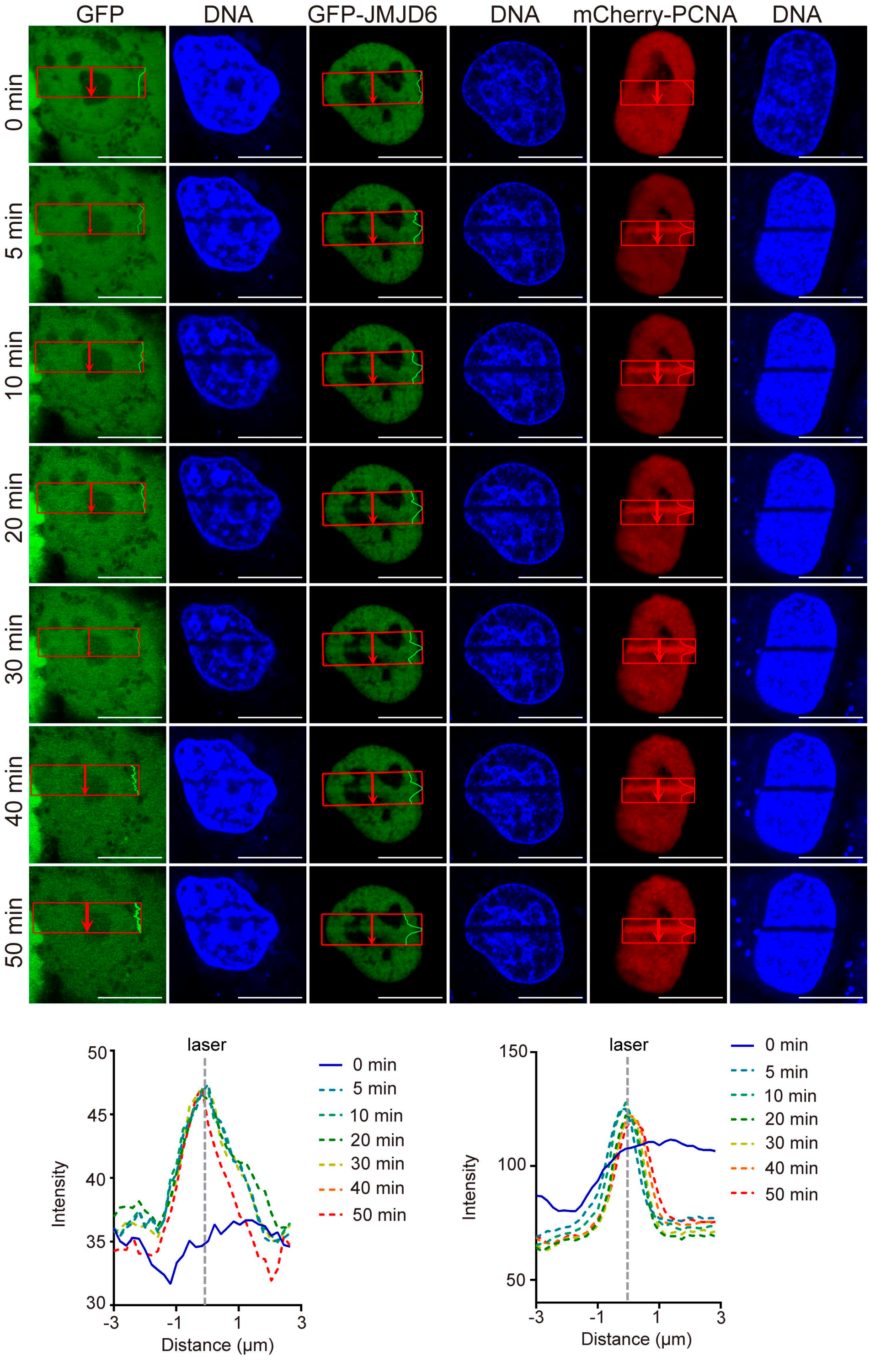

EGFP-JMJD6

mCherry-PCNA

Supplementary Figure 2

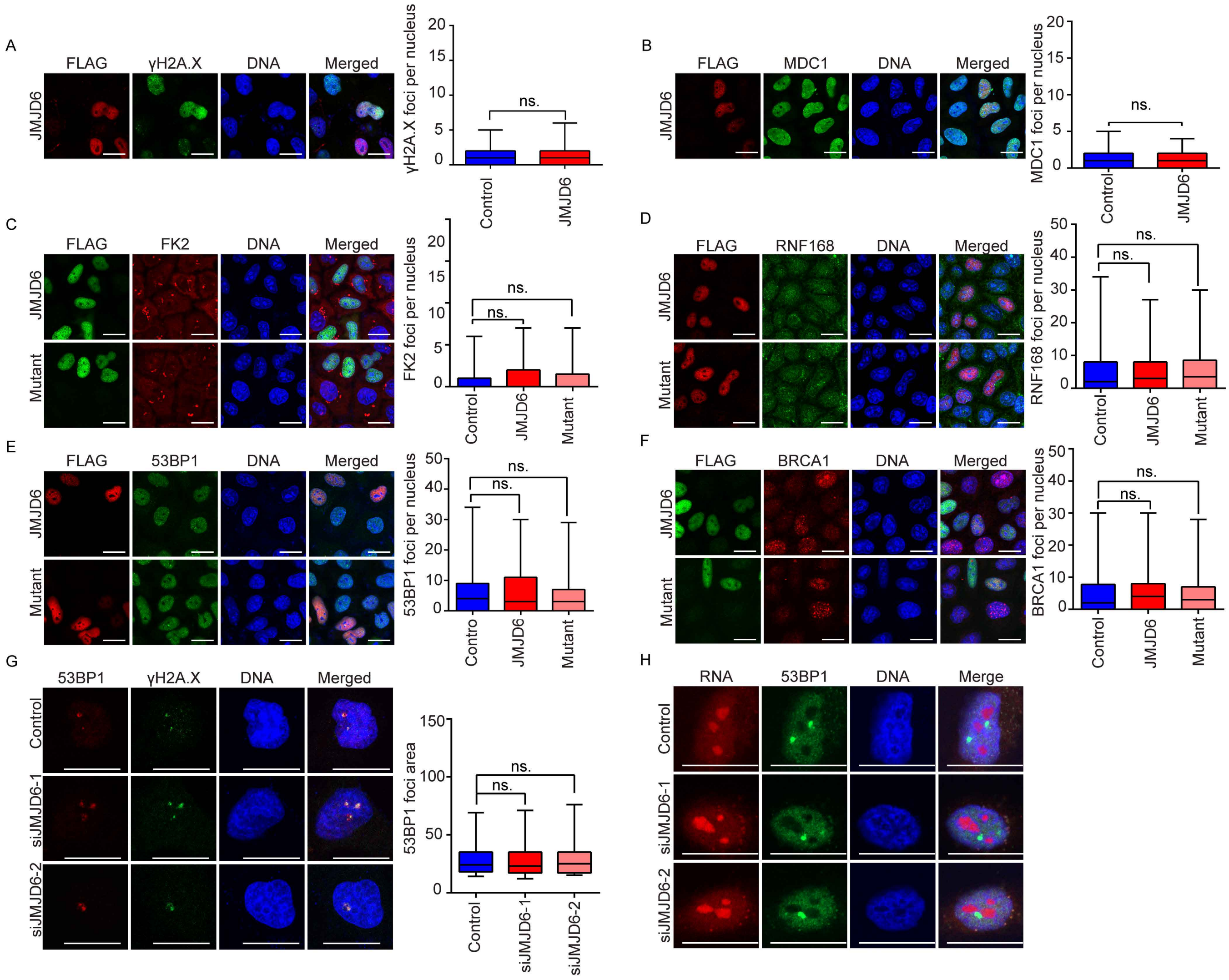

Supplementary Figure 3

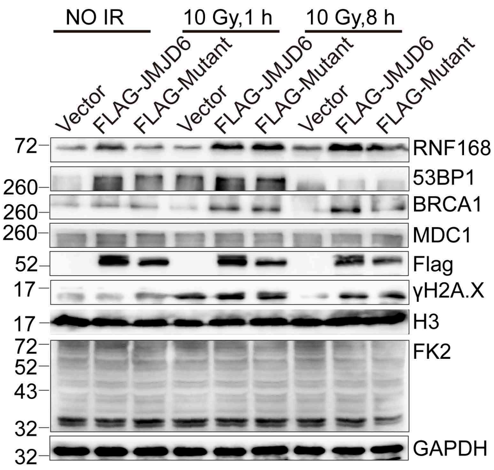

Supplementary Figure 4

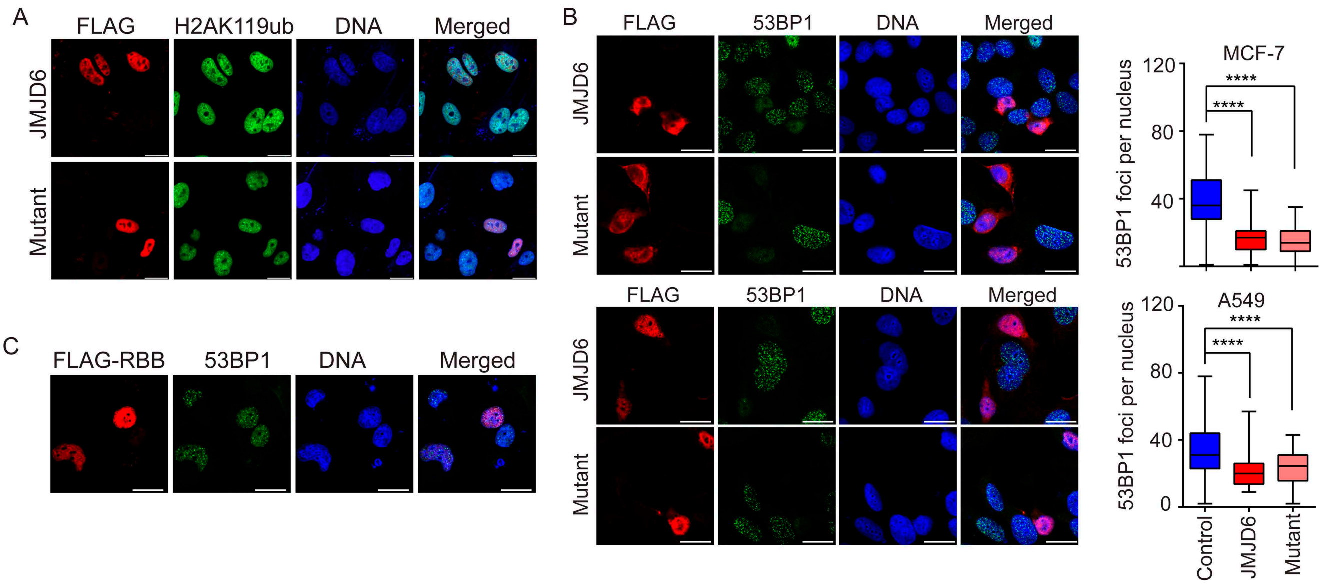

Supplementary Figure 5

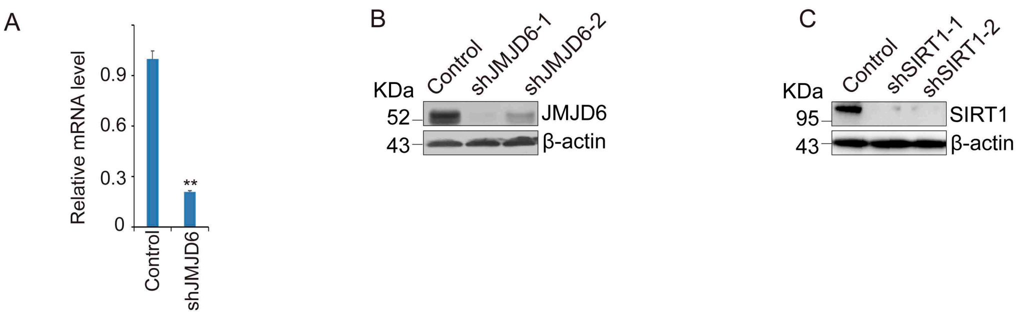

Supplementary Figure 6

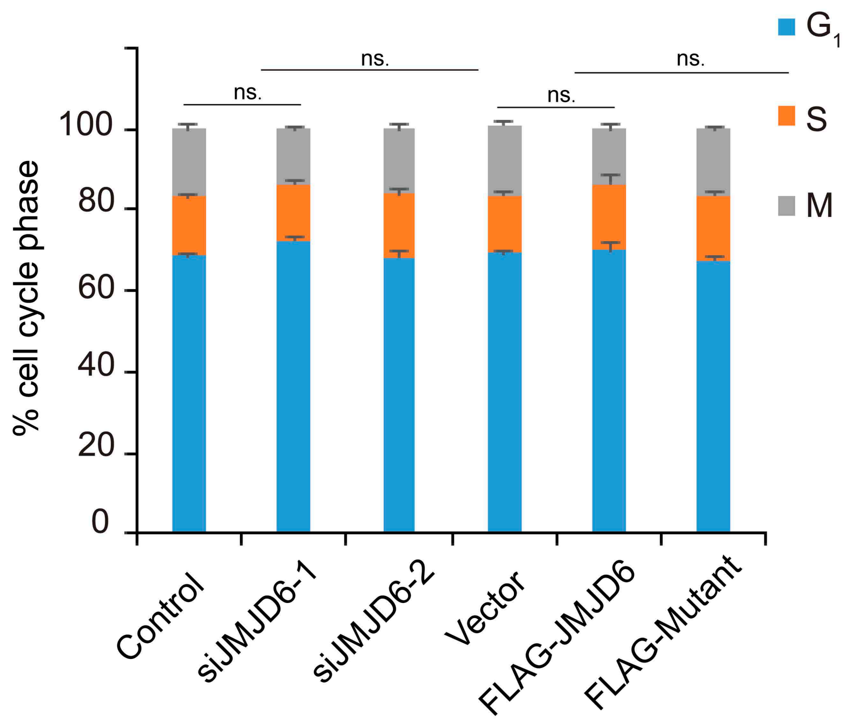

Supplementary Figure 7

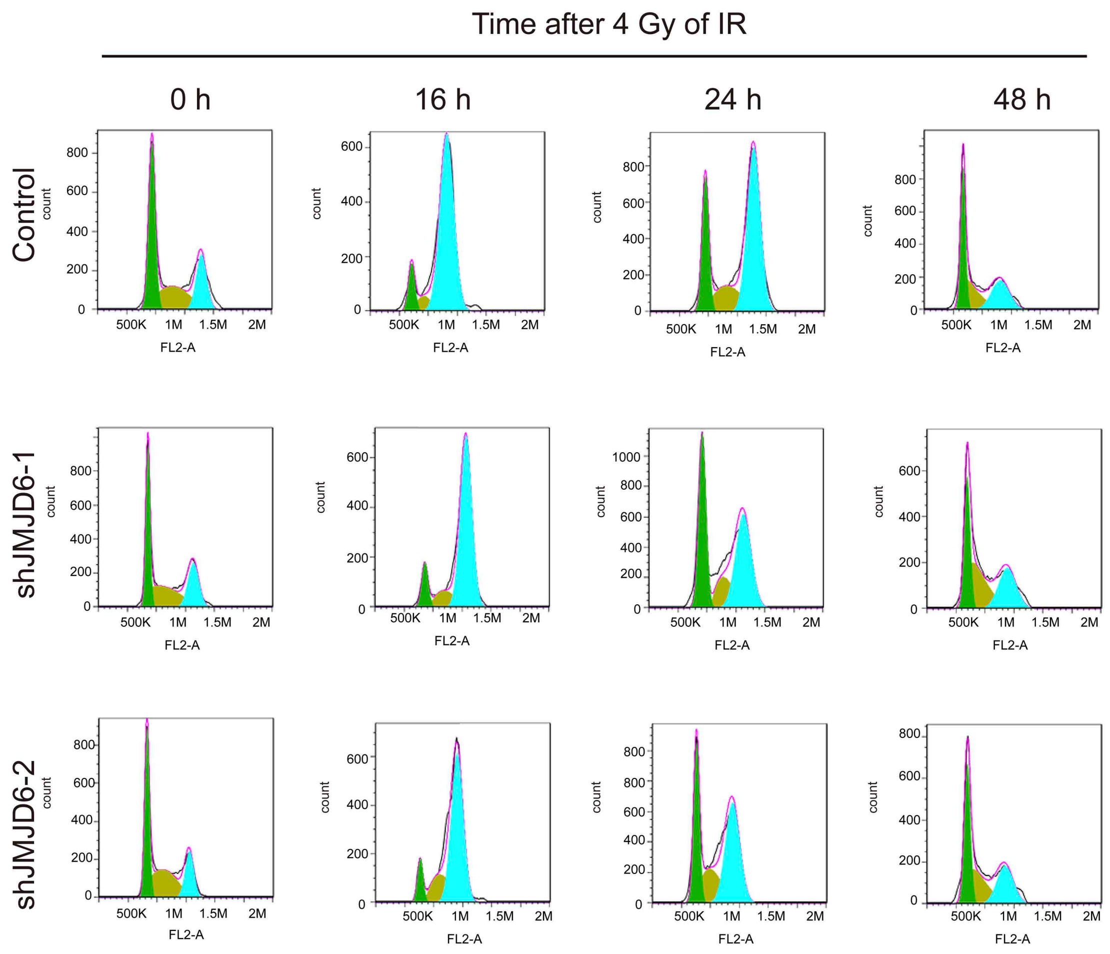

Supplementary Figure 8

Proteins	Unique peptides number	Unique peptides
LUC7L2	59	LAETQEEISAEVAAKAER
		VEQLGAEGNVEESQKVmDEVEK
		VEQLGAEGNVEESQKVMDEVEKAR
		LRVcEVcSAYLGLHDNDRR
		VEQLGAEGNVEESQKVMDEVEK
		VcKSHLLNccPHDVLSGTR
		aERVHELNEEIGK
		VHELNEEIGKLLAK
		LRVcEVcSAYLGLHDNDR
		VcEVcSAYLGLHDNDR
		AERVHELNEEIGKLLAK
		SHLLNccPHDVLSGTR
		LAETQEEISAEVAAK
		RLAETQEEISAEVAAK
		LADHFGGKLHLGFIEIR
		VEQLGAEGNVEESQK
		AERVHELNEEIGK
		sHLLNccPHDVLSGTR
		VcEVcSAYLGLHDNDRR
		lAETQEEISAEVAAK
		LLAKVEQLGAEGNVEESQK
		vcEVcSAYLGLHDNDRR
		AMLDQLMGTSRDGDTTR
		MDLGEcLKVHDLALR
		FRDQDLAScDRDR
		vEQLGAEGNVEESQK
		FRDQDLAScDR
		VEQLGAEGNVEESQKVmDEVEKAR
		VHDLALRADYEIASK
		NSMPASSFQQQK
		VMDEVEKAR
		VHELNEEIGK
		AMLDQLMGTSR
		vHELNEEIGKLLAK
		AmLDQLMGTSR
		lKFSDDRVcK
		aMLDQLMGTSR
		vHELNEEIGK
		eKLEELKR
		AmLDQLmGTSR
		nSMPASSFQQQK
		aDYEIASK
		ADYEIASK
		lHLGFIEIR
		LHLGFIEIR
		nSmPASSFQQQK
		mDLGEcLK
		mDLGEcLK
		fRDQDLAScDRDR
		mDLGEcLKVHDLALR
		vMDEVEKAR
		lADHFGGK
		eAEEVYR
		NSmPASSFQQQK
		MDLGEcLK
		VHDLALR
		vMDEVEK
		vHDLALR
		SSEEREAGEI

Supplementary Figure 9

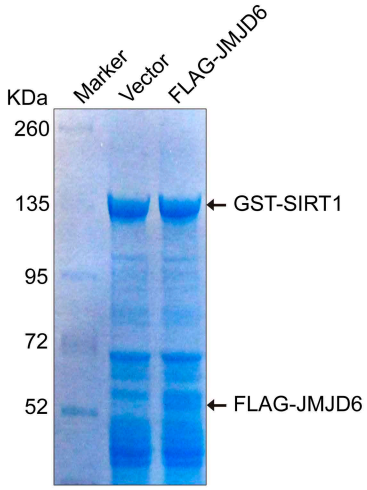

Supplementary Figure 10

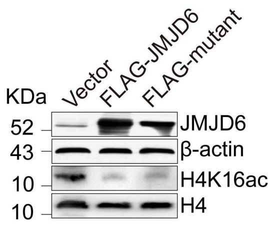

Supplementary Figure 11

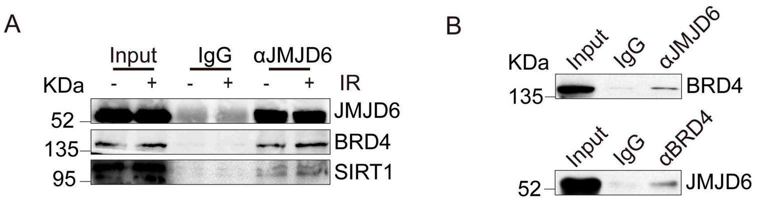

Supplementary Figure 12

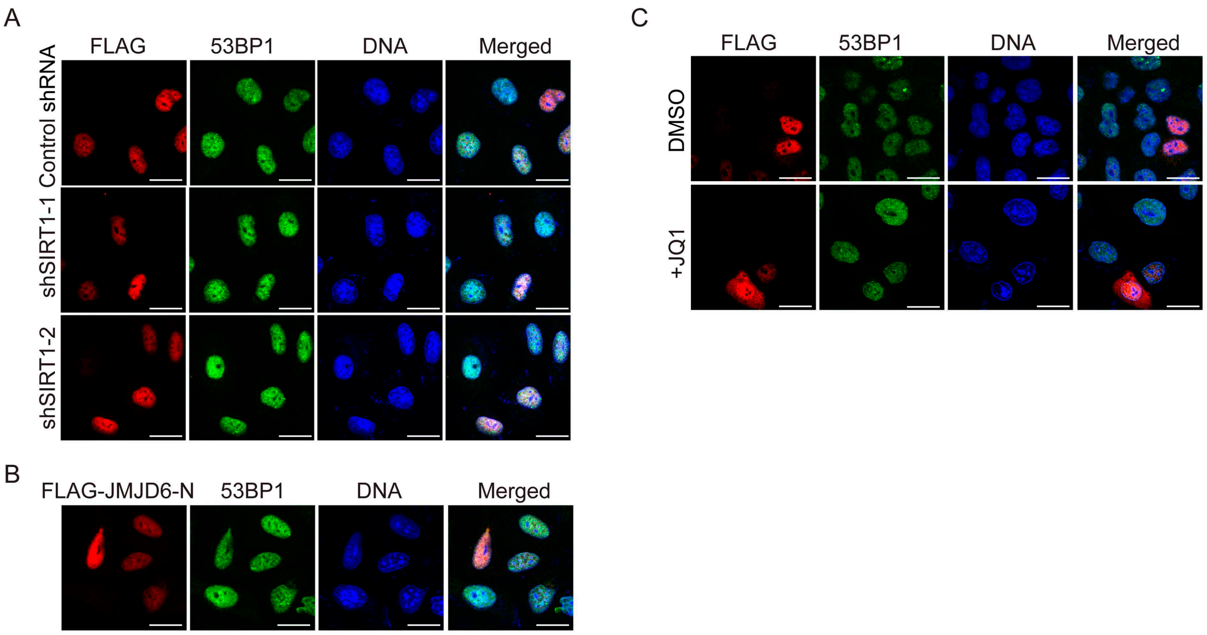

Supplementary Table 1. The detailed information of modifications on GST-SIRT1 which was incubated with control samples.

Accession	Description	Score	Coverage	Proteins	Unique Peptides	Peptides	PSMs	MW [kDa]
Q96EB6	NAD-dependent protein deacetylase sirtuin-1 OS=Homo sapiens GN=SIRT1 PE=1 SV=2 - [SIR1_HUMAN]	6551.98	78.31	3	21	56	2374	81.6
	Sequence	# PSMs	Modifications	ΔCn	q-Value	PEP	XCorr	MH+ [Da]
	AGGAGFGTDGDDQEAINEAISVK	66		0.0000	0	5.522E-15	5.99	2222.01933
	DNLLFGDEIITNGFHScESDEEDR	5	C17(Carbamidomethyl)	0.0000	0	8.452E-15	5.62	2812.19693
	eLAYLSELPPTPLHVSEDSSSPER	14	N-Term(Acetyl)	0.0000	0	6.401E-14	4.80	2695.30716
	NVESIAEQmENPDLKNVGSSTGEKNER	4	M9(Oxidation)	0.0000	0	1.037E-13	5.94	2991.39077
	IIQcHGSFATAScLIcK	8	C4(Carbamidomethyl); C13(Carbamidomethyl); C16(Carbamidomethyl)	0.0000	0	1.59E-13	5.84	1965.92900
	cPADEPLAImKPEIVFFGENLPEQFHR	117	C1(Carbamidomethyl); M10(Oxidation)	0.0000	0	2.609E-13	5.59	3200.55166
	AGGAGFGTDGDDQEAINEAISVKQEVTDMNYPSNKS	1		0.0000	0	4.332E-13	6.01	3715.66477
	cPADEPLAIMKPEIVFFGENLPEQFHR	47	C1(Carbamidomethyl)	0.0000	0	5.383E-13	6.17	3184.54897
	IIVLTGAGVSVScGIPDFR	20	C13(Carbamidomethyl)	0.0000	0	9.466E-13	4.69	1961.04985
	TQKELAYLSELPPTPLHVSEDSSSPER	1		0.0000	0	1.438E-12	6.55	3010.50380
	AMKYDKDEVDLLIVIGSSLK	6		0.0000	0	1.856E-12	5.56	2237.20786
	SNDDLDVSESKGcMEEKPQEVQTSR	4	C13(Carbamidomethyl)	0.0000	0	1.754E-11	4.00	2868.25185
	vRPVALIPSSIPHEVPQILINR	10	N-Term(Acetyl)	0.0000	0	2.04E-11	5.36	2490.45473
	AmKYDKDEVDLLIVIGSSLK	25	M2(Oxidation)	0.0000	0	3.591E-11	4.39	2253.20157

	dNLLFGDEIITNGFH ScESDEEDR	1	N-Term(Acetyl); C17(Carbamidomethyl)	0.0000	0	4.893E-11	4.35	2854.2 0041
	nYTQNIDTLEQVAGI QR	22	N-Term(Acetyl)	0.0000	0	1.149E-10	4.76	2004.9 9126
	YDKDEVDLLIVIGS SLK	64		0.0000	0	2.067E-10	5.84	1907.0 3594
	SNDDLDVSESKGcm EEKPQEVQTSR	10	C13(Carbamidomethyl); M14(Oxidation)	0.0000	0	2.323E-10	5.94	2884.2 5283
	nVESIAEQmENPDL K	17	N-Term(Acetyl); M9(Oxidation)	0.0000	0	2.386E-10	4.00	1774.8 0925
	NVESIAEQMENPDL KNVGSSTGEK	1		0.0000	0	2.547E-10	3.78	2576.2 1939
	AGGAGFGTDGDDQ EAINEAISVKQEVT DmNYPSNKS	2	M29(Oxidation)	0.0000	0	3.371E-10	6.21	3731.6 5346
	IGPYTFVQQHLmIG TDPR	39	M12(Oxidation)	0.0000	0	5.366E-10	3.14	2089.0 5278
	cPADEPLAImKPEIV FFGENLPEQFHR	6	N-Term(Acetyl); C1(Carbamidomethyl); M10(Oxidation)	0.0000	0	7.096E-10	2.60	3242.5 4880
	GcMEEKPQEVQTSR	23	C2(Carbamidomethyl)	0.0000	0	1.194E-09	4.07	1678.7 4858
	NVESIAEQMENPDL K	11		0.0000	0	1.246E-09	4.41	1716.8 0596
	TSPPDSSVIVTLLDQ AAK	207		0.0000	0	1.381E-09	4.44	1841.9 8284
	VRPVALIPSSIPHEV PQILINR	195		0.0000	0	1.423E-09	4.96	2448.4 4160
	LAVDFPDLPDPQAm FDIEYFR	2	M14(Oxidation)	0.0000	0	1.865E-09	4.24	2515.1 7990
	tSPPDSSVIVTLLDQ AAK	19	N-Term(Acetyl)	0.0000	0	2.107E-09	3.76	1883.9 9297
	EPPLADNLYDEDDD DEGEEEEEAAAAAI GYR	32		0.0000	0	3.266E-09	5.06	3411.4 0757
	KIIVLTGAGVSVScG IPDFR	2	C14(Carbamidomethyl)	0.0000	0	6.128E-09	5.06	2089.1 4604
	eAEAEAAAAGGEQ EAQATAAAGEGDN GPGLQGPSR	5	N-Term(Acetyl)	0.0000	0	6.841E-09	6.04	3280.4 5083
	LAVDFPDLPDPQAM FDIEYFR	3		0.0000	0	3.808E-08	2.80	2499.1 8583
	NVESIAEQmENPDL K	31	M9(Oxidation)	0.0000	0	6.453E-08	3.82	1732.7 9827

	FIALSDKEGKLLR	4		0.0000	0	7.361E-08	3.62	1489.87241
	RKDINTIEDAVK	26		0.0000	0	1.181E-07	4.35	1401.76856
	AGGAGFGTDGDDQEAINEAISVKQEVTDmNYPSNK	2	M29(Oxidation)	0.0000	0	1.284E-07	4.22	3644.62856
	IGPYTFVQQHLMIGTDPR	27		0.0000	0	1.859E-07	4.78	2073.05596
	rLDGNQYLFLPPNR	22	N-Term(Acetyl)	0.0000	0	2.21E-07	4.38	1744.91178
	gcMEEKPQEVQTSR	4	N-Term(Acetyl); C2(Carbamidomethyl)	0.0000	0	2.312E-07	3.50	1720.76218
	qEVTDMNYPSNK	9	N-Term(Acetyl)	0.0000	0	5.384E-07	2.99	1467.63909
	mADEAALALQPGGSPSAAGADR	4	M1(Oxidation)	0.0000	0	5.791E-07	4.89	2071.96708
	gcmEEKPQEVQTSR	6	N-Term(Acetyl); C2(Carbamidomethyl); M3(Oxidation)	0.0000	0	5.947E-07	2.80	1736.75425
	aMKYDKDEVDLLIVIGSSLK	1	N-Term(Acetyl)	0.0000	0	7.671E-07	4.60	2279.21767
	QEVTDmNYPSNK	19	M6(Oxidation)	0.0000	0	1.397E-06	3.62	1441.62517
	ELAYLSELPPTPLHVSEDSSSPER	123		0.0000	0	1.442E-06	5.51	2653.29653
	QEVTDMNYPSNK	10		0.0000	0	1.798E-06	3.61	1425.62944
	MADEAALALQPGGSPSAAGADR	3		0.0000	0	1.818E-06	3.74	2055.97501
	EAEAEAAAAGGEQEAQATAAAGEGDNGPGLQGPSR	47		0.0000	0	2.941E-06	6.90	3238.44007
	LSEITEKPPR	88		0.0000	0	3.34E-06	3.64	1169.65180
	GcmEEKPQEVQTSR	10	C2(Carbamidomethyl); M3(Oxidation)	0.0000	0	3.41E-06	3.44	1694.74985
	KDINTIEDAVK	16		0.0000	0	3.656E-06	4.92	1245.66619
	nVESIAEQMENPDLK	6	N-Term(Acetyl)	0.0000	0	4.776E-06	3.81	1758.81767
	EAASSPAGEPLRK	9		0.0000	0	4.893E-06	2.93	1312.68454
	yDKDEVDLLIVIGSS	2	N-Term(Acetyl)	0.0000	0	5.01E-06	3.00	1949.0

	LK							4288
	SPGEPGGAAPEREVPAAAR	3		0.0000	0	5.331E-06	3.73	1818.90818
	qEVTDmNYPSNK	5	N-Term(Acetyl); M6(Oxidation)	0.0000	0	5.845E-06	2.91	1483.63445
	NVGSSTGEKNER	3		0.0000	0	8.062E-06	2.15	1277.60776
	SNDDLDVSESK	13		0.0000	0	1.035E-05	3.16	1208.52800
	LDGNQYLFLPPNR	22		0.0000	0	1.268E-05	3.44	1546.79827
	kDINTIEDAVK	7	N-Term(Acetyl)	0.0000	0	1.384E-05	4.73	1287.67546
	gcPGAAAAALWR	2	N-Term(Acetyl); C2(Carbamidomethyl)	0.0000	0	1.741E-05	2.22	1242.60503
	qEVTDMNYPSNKS	2	N-Term(Acetyl)	0.0000	0	0.0000188	3.40	1554.67131
	FAKEIYPGQFQPSLcHK	1	C15(Carbamidomethyl)	0.0000	0	1.888E-05	3.07	2050.02097
	aGGAGFGTDGDDQEAINEAISVK	14	N-Term(Acetyl)	0.0000	0	2.217E-05	4.90	2264.02567
	QEVTDMNYPSNKS	11		0.0000	0	2.957E-05	3.35	1512.66106
	eAASSPAGEPLRK	12	N-Term(Acetyl)	0.0000	0	3.123E-05	3.76	1354.69434
	fIALSDKEGK	10	N-Term(Acetyl)	0.0000	0	3.143E-05	2.36	1149.61457
	rKDINTIEDAVK	6	N-Term(Acetyl)	0.0000	0	3.849E-05	3.83	1443.77973
	qEVTDmNYPSNKS	5	N-Term(Acetyl); M6(Oxidation)	0.0000	0	5.399E-05	2.83	1570.66606
	RLDGNQYLFLPPNR	127		0.0000	0	5.715E-05	4.74	1702.90427
	GcPGAAAAALWR	8	C2(Carbamidomethyl)	0.0000	0	6.608E-05	3.79	1200.59269
	SPGEPGGAAPER	9		0.0000	0	8.366E-05	2.74	1124.53276
	FIALSDKEGK	39		0.0000	0	0.0000856	2.55	1107.60376
	lSEITEKPPR	43	N-Term(Acetyl)	0.0000	0	0.0001108	3.35	1211.66130
	amKYDKDEVDLLIVIGSSLK	6	N-Term(Acetyl); M2(Oxidation)	0.0000	0	0.0001112	3.60	2295.21132

	sNDDLDVSESK	15	N-Term(Acetyl)	0.0000	0	0.0001151	3.00	1250.53691
	eAASSPAGEPLR	13	N-Term(Acetyl)	0.0000	0	0.0002556	3.41	1226.59966
	YKVDcEAVR	16	C5(Carbamidomethyl)	0.0000	0	0.0002562	3.14	1139.54999
	NYTQNIDTLEQVAGIQR	95		0.0000	0	0.000282	4.61	1962.98403
	EAASSPAGEPLR	30		0.0000	0	0.0003917	3.33	1184.59075
	iGPYTFVQQHLMIGTDPR	2	N-Term(Acetyl)	0.0000	0	0.0004022	1.54	2115.06841
	DINTIEDAVK	100		0.0000	0	0.0006895	2.76	1117.57585
	LLQEcKK	2	C5(Carbamidomethyl)	0.0000	0	0.0007237	2.14	918.50699
	DNLLFGDEIITNGFHScESDEEDRASHASSSDWTPRPR	1	C17(Carbamidomethyl)	0.0000	0	0.000756	5.09	4347.90113
	GDIFNQVVPR	92		0.0000	0	0.0008058	3.18	1144.61057
	dINTIEDAVK	28	N-Term(Acetyl)	0.0000	0	0.0008466	2.74	1159.58367
	lDGNQYLFLPPNR	2	N-Term(Acetyl)	0.0000	0	0.001448	3.47	1588.81235
	rDGPGLER	2	N-Term(Acetyl)	0.0000	0	0.00237	2.46	941.47887
	QEVTDmNYPSNKS	20	M6(Oxidation)	0.0000	0	0.003457	3.27	1528.65300
	iGPYTFVQQHLmIGTDPR	6	N-Term(Acetyl); M12(Oxidation)	0.0000	0	0.00352	2.03	2131.06125
	DPRPFFK	11		0.0000	0	0.004142	2.43	906.48099
	DGIYAR	4		0.0000	0	0.005339	1.68	694.35124
	lccNPVK	8	N-Term(Acetyl); C2(Carbamidomethyl); C3(Carbamidomethyl)	0.0000	0	0.005528	1.94	932.43260
	gDIFNQVVPR	15	N-Term(Acetyl)	0.0000	0	0.00724	3.08	1186.61991
	EIYPGQFQPSLcHK	27	C12(Carbamidomethyl)	0.0000	0	0.008582	3.34	1703.82155
	eIYPGQFQPSLcHK	7	N-Term(Acetyl); C12(Carbamidomethyl)	0.0000	0	0.01277	2.29	1745.82694

	fIALSDK	2	N-Term(Acetyl)	0.0000	0.001	0.018	1.71	835.45537
	FIALSDK	13		0.0000	0.001	0.02786	2.13	793.44560
	kDPRPFFK	3	N-Term(Acetyl)	0.0000	0.002	0.04568	1.63	1076.58753
	KDPRPFFKFAK	1		0.0000	0.003	0.094	1.45	1380.77834

Supplementary Table 2. The detailed information of modifications on GST-SIRT1 which was incubated with FLAG-JMJD6.

Accession	Description	Score	Coverage	Proteins	Unique Peptides	Peptides	PSMs	MW [kDa]
Q96EB6	NAD-dependent protein deacetylase sirtuin-1 OS=Homo sapiens GN=SIRT1 PE=1 SV=2 - [SIR1_HUMAN]	7850.79	84.47	4	63	63	2737	81.6
	Sequence	# PSMs	Modifications	ΔCn	q-Value	PEP	XCorr	MH+ [Da]
	AMKYDKDEVDLLIVIGSSLK	30		0.0000	0	7.102E-07	6.58	2237.20293
	SNDDLDVSESKGcMEEKPQEVQTSR	4	C13(Carbamidomethyl)	0.0000	0	1.21E-06	5.87	2868.25980
	TQKELAYLSELPPTPLHVSEDSSSPER	2		0.0000	0	4.352E-06	7.09	3010.50966
	AGGAGFGTDGDDQEAINEAISVKQEVTDMNYPSNK	2		0.0000	0	4.888E-06	6.44	3628.61643
	NVESIAEQMENPDLKNVGSSTGEK	1		0.0000	0	6.557E-06	4.26	2576.21372
	DNLLFGDEIITNGFHScESDEEDR	4	C17(Carbamidomethyl)	0.0000	0	6.767E-06	6.08	2812.19217
	IIQcHGSFATAScLIcK	8	C4(Carbamidomethyl); C13(Carbamidomethyl); C16(Carbamidomethyl)	0.0000	0	0.0000106	6.46	1965.92974
	YDKDEVDLLIVIGSSLK	97		0.0000	0	1.531E-05	5.55	1907.03386
	IGPYTFVQQHLmIGTDPR	18	M12(Oxidation)	0.0000	0	2.659E-05	3.79	2089.05132
	EAEAEAAAGGEQ	59		0.0000	0	2.921E-05	6.80	3238.4

	EAQATAAAGEGDN GPGLQGPSR							4375
	ELAYLSELPPTPLH VSEDSSSPER	113		0.0000	0	3.755E-05	5.78	2653.2 8847
	cPADEPLAIMKPEIV FFGENLPEQFHR	83	C1(Carbamidomethyl)	0.0000	0	4.034E-05	5.76	3184.5 5044
	NVESIAEQmENPDL KNVGSSTGEKNER	2	M9(Oxidation)	0.0000	0	5.548E-05	6.36	2991.3 9069
	VRPVALIPSSIPHEV PQILINR	185		0.0000	0	6.274E-05	4.98	2448.4 4450
	nYTQNIDTLEQVAGI QR	26	N-Term(Acetyl)	0.0000	0	7.584E-05	4.82	2004.9 9346
	eAEAEAAAAGGEQ EAQATAAAGEGDN GPGLQGPSR	8	N-Term(Acetyl)	0.0000	0	7.598E-05	6.13	3280.4 5571
	eLAYLSELPPTPLHV SEDSSSPER	16	N-Term(Acetyl)	0.0000	0	8.065E-05	4.48	2695.3 0057
	IIVLTGAGVSVScGI PDFR	127	C13(Carbamidomethyl)	0.0000	0	8.116E-05	4.74	1961.0 4839
	yDKDEVDLLIVIGSS LK	12	N-Term(Acetyl)	0.0000	0	9.433E-05	4.63	1949.0 4228
	KIIVLTGAGVSVScG IPDFR	27	C14(Carbamidomethyl)	0.0000	0	0.000170 8	5.49	2089.1 5068
	IGPYTFVQQHLMIG TDPR	88		0.0000	0	0.000186 2	4.88	2073.0 5351
	vRPVALIPSSIPHEVP QILINR	16	N-Term(Acetyl)	0.0000	0	0.000216 1	4.96	2490.4 4887
	lAVDFPDLPDPQAM FDIEYFR	7	N-Term(Acetyl)	0.0000	0	0.000250 4	3.98	2541.1 9571
	SNDDLDVSESKGcm EEKPQEVQTSR	8	C13(Carbamidomethyl); M14(Oxidation)	0.0000	0	0.000276 9	6.49	2884.2 5234
	aGGAGFGTDGDDQ EAINEAISVK	17	N-Term(Acetyl)	0.0000	0	0.000296 9	4.89	2264.0 3301
	GcMEEKPQEVQTSR	37	C2(Carbamidomethyl)	0.0000	0	0.000297 2	4.23	1678.7 4761
	LAVDFPDLPDPQAM FDIEYFRK	2		0.0000	0	0.000326 9	3.84	2627.2 8037
	nVESIAEQmENPDL K	17	N-Term(Acetyl); M9(Oxidation)	0.0000	0	0.000337 1	4.37	1774.8 1511
	NVESIAEQMENPDL K	47		0.0000	0	0.000366	4.15	1716.8 0828
	RLDGNQYLFLPPNR	125		0.0000	0	0.000423 1	4.92	1702.8 9946
	nVESIAEQMENPDL	17	N-Term(Acetyl)	0.0000	0	0.000465	4.54	1758.8

	K					1		2097
	iIVLTGAGVSVScGIPDFR	2	N-Term(Acetyl); C13(Carbamidomethyl)	0.0000	0	0.0005669	3.92	2003.06266
	NVGSSTGEKNER	4		0.0000	0	0.0006516	2.50	1277.60776
	tSPPDSSVIVTLLDQAAK	30	N-Term(Acetyl)	0.0000	0	0.0006622	4.18	1883.99321
	AGGAGFGTDGDDQEAINEAISVK	97		0.0000	0	0.0007068	5.96	2222.02018
	EPPLADNLYDEDDDDEGEEEEEAAAAAIGYR	32		0.0000	0	0.0007424	5.04	3411.39760
	LAVDFPDLPDPQAMFDIEYFR	16		0.0000	0	0.0008095	3.81	2499.18528
	DEVDLLIVIGSSLK	3		0.0000	0	0.0009699	3.72	1500.84795
	NVESIAEQmENPDLK	26	M9(Oxidation)	0.0000	0	0.001004	3.36	1732.80217
	AmKYDKDEVDLLIVIGSSLK	61	M2(Oxidation)	0.0000	0	0.001062	4.27	2253.20376
	TSPPDSSVIVTLLDQAAK	174		0.0000	0	0.001124	4.37	1841.97966
	gcMEEKPQEVQTSR	12	N-Term(Acetyl); C2(Carbamidomethyl)	0.0000	0	0.001257	3.58	1720.75835
	lSEITEKPPR	64	N-Term(Acetyl)	0.0000	0	0.001281	3.30	1211.66132
	GcmEEKPQEVQTSR	15	C2(Carbamidomethyl); M3(Oxidation)	0.0000	0	0.001313	3.71	1694.74527
	LAVDFPDLPDPQAmFDIEYFR	12	M14(Oxidation)	0.0000	0	0.001437	3.73	2515.18045
	gcmEEKPQEVQTSR	7	N-Term(Acetyl); C2(Carbamidomethyl); M3(Oxidation)	0.0000	0	0.001554	3.07	1736.75663
	EPLPHLHFDVELLGDcDVIINELcHR	2	C16(Carbamidomethyl); C24(Carbamidomethyl)	0.0000	0	0.001601	4.66	3140.51748
	eAASSPAGEPLRK	17	N-Term(Acetyl)	0.0000	0	0.001672	3.76	1354.69233
	KDINTIEDAVK	12		0.0000	0	0.001713	5.02	1245.66545
	iGPYTFVQQHLMIGTDPR	12	N-Term(Acetyl)	0.0000	0	0.001907	3.65	2115.06890
	kDINTIEDAVK	8	N-Term(Acetyl)	0.0000	0	0.002158	4.71	1287.67571
	rKDINTIEDAVK	10	N-Term(Acetyl)	0.0000	0	0.002459	3.38	1443.7

								8009
	AGGAGFGTDGDDQEAINEAISVKQEVTDmNYPSNKS	1	M29(Oxidation)	0.0000	0	0.002844	4.28	3731.63857
	mADEAALALQPGGSPSAAGADR	2	M1(Oxidation)	0.0000	0	0.002868	3.63	2071.96946
	LSEITEKPPR	73		0.0000	0	0.003301	3.69	1169.65125
	sNDDLDVSESK	21	N-Term(Acetyl)	0.0000	0	0.003383	2.93	1250.53703
	rLDGNQYLFLPPNR	55	N-Term(Acetyl)	0.0000	0	0.003384	4.38	1744.91050
	LAVDFPDLPDPQAmFDIEYFRK	1	M14(Oxidation)	0.0000	0	0.003551	4.14	2643.27610
	lAVDFPDLPDPQAmFDIEYFR	3	N-Term(Acetyl); M14(Oxidation)	0.0000	0	0.003745	4.63	2557.18540
	FIALSDKEGKLLR	5		0.0000	0	0.003962	3.57	1489.87259
	sNDDLDVSESKGcmEEKPQEVQTSR	3	N-Term(Acetyl); C13(Carbamidomethyl); M14(Oxidation)	0.0000	0	0.004237	3.58	2926.25991
	aMKYDKDEVDLLIVIGSSLK	2	N-Term(Acetyl)	0.0000	0	0.00466	4.18	2279.21645
	MADEAALALQPGGSPSAAGADR	1		0.0000	0	0.005578	4.17	2055.97446
	QEVTDmNYPSNK	14	M6(Oxidation)	0.0000	0	0.00561	3.52	1441.62370
	NVGSSTGEKNERTSVAGTVR	2		0.0000	0	0.005975	4.49	2049.02724
	LDGNQYLFLPPNR	20		0.0000	0	0.006403	3.31	1546.79875
	GcPGAAAAALWR	6	C2(Carbamidomethyl)	0.0000	0	0.006884	3.57	1200.59186
	EAASSPAGEPLRK	8		0.0000	0	0.007069	2.96	1312.68463
	YKVDcEAVR	20	C5(Carbamidomethyl)	0.0000	0	0.007615	3.66	1139.55036
	QEVTDMNYPSNK	14		0.0000	0	0.007645	3.64	1425.62871
	SNDDLDVSESK	11		0.0000	0	0.008592	3.34	1208.52678
	qEVTDMNYPSNK	4	N-Term(Acetyl)	0.0000	0	0.008661	3.20	1467.63652
	nVGSSTGEKNER	1	N-Term(Acetyl)	0.0000	0	0.009582	1.48	1319.6

								1774
	qEVTDMNYPSNKS	6	N-Term(Acetyl)	0.0000	0	0.01017	3.21	1554.67266
	lDGNQYLFLPPNR	5	N-Term(Acetyl)	0.0000	0	0.01022	3.81	1588.81059
	FIALSDKEGK	38		0.0000	0	0.01042	2.86	1107.60349
	SPGEPGGAAPER	6		0.0000	0	0.01082	2.99	1124.53252
	IGPYTFVQQHLMIGTDPRTILK	1		0.0000	0	0.01101	3.31	2528.36709
	qEVTDmNYPSNK	4	N-Term(Acetyl); M6(Oxidation)	0.0000	0	0.01165	2.63	1483.63078
	QEVTDMNYPSNKS	16		0.0000	0	0.01205	3.57	1512.66167
	aSHASSSDWTPRPR	5	N-Term(Acetyl)	0.0000	0	0.01216	3.81	1596.74904
	NYTQNIDTLEQVAGIQR	62		0.0000	0	0.01244	4.80	1962.99111
	qEVTDmNYPSNKS	4	N-Term(Acetyl); M6(Oxidation)	0.0000	0	0.01388	2.84	1570.66789
	cPADEPLAIMKPEIVFFGENLPEQFHR	1	N-Term(Acetyl); C1(Carbamidomethyl)	0.0000	0	0.01531	3.20	3226.56232
	eAASSPAGEPLR	16	N-Term(Acetyl)	0.0000	0	0.01698	3.39	1226.59868
	GDIFNQVVPR	82		0.0000	0	0.01698	3.12	1144.60865
	iGPYTFVQQHLmIGTDPR	9	N-Term(Acetyl); M12(Oxidation)	0.0000	0	0.01754	2.30	2131.06206
	EIYPGQFQPSLcHK	26	C12(Carbamidomethyl)	0.0000	0	0.02114	3.00	1703.81778
	gDIFNQVVPR	21	N-Term(Acetyl)	0.0000	0	0.0225	2.84	1186.61894
	rDGPGLER	4	N-Term(Acetyl)	0.0000	0	0.02274	2.65	941.47936
	eIYPGQFQPSLcHK	14	N-Term(Acetyl); C12(Carbamidomethyl)	0.0000	0	0.02371	2.34	1745.82914
	amKYDKDEVDLLIVIGSSLK	2	N-Term(Acetyl); M2(Oxidation)	0.0000	0	0.02382	3.14	2295.22085
	tSVAGTVR	3	N-Term(Acetyl)	0.0000	0	0.0239	1.51	832.45079
	gcPGAAAAALWR	1	N-Term(Acetyl); C2(Carbamidomethyl)	0.0000	0	0.02707	1.84	1242.60381

	DINTIEDAVK	71		0.0000	0	0.03046	2.86	1117.57146
	RKDINTIEDAVK	21		0.0000	0	0.03197	4.32	1401.76746
	fIALSDKEGK	15	N-Term(Acetyl)	0.0000	0	0.03202	2.64	1149.61338
	dINTIEDAVK	27	N-Term(Acetyl)	0.0000	0	0.03599	2.66	1159.58281
	EAASSPAGEPLR	30		0.0000	0	0.03927	3.22	1184.58867
	LLQEcKK	1	C5(Carbamidomethyl)	0.0000	0	0.04009	2.16	918.50672
	DPRPFFK	9		0.0000	0	0.04576	2.53	906.48081
	cPADEPLAImKPEIVFFGENLPEQFHR	5	N-Term(Acetyl); C1(Carbamidomethyl); M10(Oxidation)	0.0000	0	0.05126	2.97	3242.58144
	SPGEPGGAAPEREVPAAAR	1		0.0000	0	0.06035	2.71	1818.90818
	dPRPFFK	1	N-Term(Acetyl)	0.0000	0	0.06988	1.71	948.49152
	sPGEPGGAAPER	1	N-Term(Acetyl)	0.0000	0	0.07272	1.46	1166.54314
	QEVTDmNYPSNKS	17	M6(Oxidation)	0.0000	0	0.07735	2.97	1528.65801
	SRDGIYAR	3		0.0000	0	0.09829	2.61	937.48363
	lccNPVK	8	N-Term(Acetyl); C2(Carbamidomethyl); C3(Carbamidomethyl)	0.0000	0	0.09868	1.95	932.43218
